# Similarities and differences in the localization, trafficking, and function of P-glycoprotein in *MDR1*-EGFP-transduced rat versus human brain capillary endothelial cell lines

**DOI:** 10.1186/s12987-021-00266-z

**Published:** 2021-08-03

**Authors:** Birthe Gericke, Saskia Borsdorf, Inka Wienböker, Andreas Noack, Sandra Noack, Wolfgang Löscher

**Affiliations:** 1 Department of Pharmacology, Toxicology, and Pharmacy, University of Veterinary Medicine Hannover, Bünteweg 17, 30559 Hannover, Germany; 2 Center for Systems Neuroscience, Hannover, Germany; 3 Department of Trauma Surgery, Hannover Medical School, Hannover, Germany

**Keywords:** Blood–brain barrier, Lysosomal trapping, Drug resistance, Doxorubicin, Species differences

## Abstract

**Background:**

In vitro models based on brain capillary endothelial cells (BCECs) are among the most versatile tools in blood–brain barrier research for testing drug penetration into the brain and how this is affected by efflux transporters such as P-glycoprotein (Pgp). However, compared to freshly isolated brain capillaries or primary BCECs, the expression of Pgp in immortalized BCEC lines is markedly lower, which prompted us previously to transduce the widely used human BCEC line hCMEC/D3 with a doxycycline-inducible *MDR1*-EGFP fusion plasmid. The EGFP-labeled Pgp in these cells allows studying the localization and trafficking of the transporter and how these processes are affected by drug exposure. Here we used this strategy for the rat BCEC line RBE4 and performed a face-to-face comparison of RBE4 and hCMEC/D3 wild-type (WT) and *MDR1*-EGFP transduced cells.

**Methods:**

*MDR1*-EGFP-transduced variants were derived from WT cells by lentiviral transduction, using an *MDR1*-linker-EGFP vector. Localization, trafficking, and function of Pgp were compared in WT and *MDR1*-EGFP transduced cell lines. Primary cultures of rat BCECs and freshly isolated rat brain capillaries were used for comparison.

**Results:**

All cells exhibited typical BCEC morphology. However, significant differences were observed in the localization of Pgp in that RBE4-*MDR1*-EGFP cells expressed Pgp primarily at the plasma membrane, whereas in hCMEC/D3 cells, the Pgp-EGFP fusion protein was visible both at the plasma membrane and in endolysosomal vesicles. Exposure to doxorubicin increased the number of Pgp-EGFP-positive endolysosomes, indicating a lysosomotropic effect. Furthermore, lysosomal trapping of doxorubicin was observed, likely contributing to the protection of the cell nucleus from damage. In cocultures of WT and *MDR1*-EGFP transduced cells, intercellular Pgp-EGFP trafficking was observed in RBE4 cells as previously reported for hCMEC/D3 cells. Compared to WT cells, the *MDR1*-EGFP transduced cells exhibited a significantly higher expression and function of Pgp. However, the junctional tightness of WT and *MDR1*-EGFP transduced RBE4 and hCMEC/D3 cells was markedly lower than that of primary BCECs, excluding the use of the cell lines for studying vectorial drug transport.

**Conclusions:**

The present data indicate that *MDR1*-EGFP transduced RBE4 cells are an interesting tool to study the biogenesis of lysosomes and Pgp-mediated lysosomal drug trapping in response to chemotherapeutic agents and other compounds at the level of the blood–brain barrier.

**Supplementary Information:**

The online version contains supplementary material available at 10.1186/s12987-021-00266-z.

## Background

P-glycoprotein (Pgp), the product of the multidrug resistance 1 (*MDR1*) or *ABCB1* gene, is an ATP-dependent efflux transporter that affects the absorption, distribution, and excretion of various clinically important drugs [1]. At the blood–brain barrier (BBB), which tightly regulates the movement of ions, molecules, and cells between the blood and the brain, Pgp is mainly located at the apical (luminal) surface of brain capillary endothelial cells (BCECs) that primarily form the BBB [2]. Through this localization, Pgp restricts or prevents brain entry of a wide variety of small lipophilic drugs, which presents a significant hurdle to the treatment of various central nervous system (CNS) diseases [1, 3–5]. Thus, the identification of compounds that are Pgp substrates is important to aid the optimization and selection of new drug candidates [6–9].

In addition to the plasma membrane, several studies suggested that Pgp is localized in the membrane of lysosomes and may mediate the active sequestration of anticancer drugs in lysosomes [10–12]. Indeed, anticancer drugs such as doxorubicin enter the lysosome either by passive diffusion along the cytoplasmic to lysosomal pH gradient (the lysosomal pH is approximately 5) or may be actively transported across the membrane by inward-turned Pgp pumps embedded in the lysosomal membrane. This so-called “drug safe house” effect of lysosomes is thought to contribute to the chemoresistance of cancer cells, but, as shown by us recently, also occurs in other cell types such as BCECs that form the BBB [13].

A variety of in vitro assays have been developed as high-throughput and low-cost alternatives to excessive animal testing for classifying compounds as Pgp substrates, including inhibition assays (based on cellular uptake of rhodamine 123 (Rho123) or calcein-AM) and functional assays (ATPase activity assay and transcellular transport assay) [6–8, 14]. For large-scale Pgp screening of new chemical entities (NCEs), often BBB surrogate models such as *MDR1*-transfected MDCKII (Madin-Darby canine kidney strain II) and LLC-PK1 (Lilly Laboratories cells—porcine kidney 1) cells or a human colon carcinoma cell line (Caco-2) are used as general barrier models, because these polarized epithelial cells form tight monolayers resulting in high transepithelial/-endothelial electrical resistance (TEER) and low paracellular permeability, thus enabling to study transcellular Pgp-mediated vectorial drug transport [14–20]. However, such simple epithelial cell models do not reflect the complexity of the BBB but exhibit different cytoarchitecture and genetically programmed molecular differences, such as the expression of specific sets of tight junction proteins and the lack of several BBB-specific transporters [15, 21]. Indeed, when directly comparing identification of Pgp substrates by BBB surrogate models vs. a triple culture of primary rat BCECs with pericytes and astrocytes, the Caco-2 and MDCK-*MDR1* models identified more Pgp drug substrates than the rat brain BBB model [22]. Furthermore, the rat brain BBB model resulted in the best correlation with in vivo drug permeability data in rodents [22]. However, primary BBB cell culture models are too costly and laborious for routine use.

This has led to the development of several immortalized BCEC lines that share many of the in vivo characteristics of the BBB [16, 17, 23–26]. The rat endothelial cell line RBE4 [27] and the human endothelial cell line hCMEC/D3 [28] are the most commonly used immortalized BCEC lines for the establishment of in vitro BBB models [23–26]. However, as all available BCEC lines, both RBE4 and hCMEC/D3 cells exhibit a relatively low junctional tightness under routine culture conditions, which is a challenge regarding their use for studying vectorial transport of small molecule compounds [23, 25, 29]. Furthermore, both cell lines exhibit markedly lower Pgp expression than rat or human primary BCECs or freshly isolated brain capillaries, which restricts the use of these immortalized cells as in vitro models for classifying compounds as Pgp substrates [29, 30]. For this reason we transduced hCMEC/D3 cells with a doxycycline-inducible *MDR1*-EGFP (enhanced green fluorescent fusion protein) fusion plasmid, which resulted in a 15-fold increase in Pgp expression and an increased efflux of the Pgp substrate Rho123 [31]. Furthermore, the EGFP-labeled Pgp expressed by these cells allowed us to study drug-induced intra- and intercellular trafficking of Pgp [13, 32] and led to the recent discovery of a novel mechanism of drug disposal at the BBB [9, 13].

Pgp is involved in a complex relationship with its host membrane environment, which modulates the various functions of the protein, including ATP hydrolysis, drug binding, and drug transport [33]. Species differences have been described both for the membrane properties of BCECs and for Pgp function [23, 34, 35], which complicates studying functional differences of Pgp in BCECs of different species. In the present study, we transduced RBE4 cells with the doxycycline-inducible *MDR1*-EGFP fusion plasmid, which allowed us to compare the localization, trafficking, and function of human Pgp in BCEC lines from two different species, resulting in interesting similarities and differences. For this purpose, with few exceptions, the experiments in wild-type (WT) and *MDR1*-EGFP transduced RBE4 and hCMEC/D3 cells were performed face-to-face. For comparison, primary cultured rat BCECs (rBCECs), freshly prepared rat brain capillaries, and, for vectorial drug transport, *MDR1*-transfected LLC-PK1 cells were used.

## Materials and methods

### Cell lines and cell culture

In this study two immortalized brain capillary endothelial cell lines were used. Rat brain endothelial (RBE4) cells were kindly provided by Prof. Francoise Roux (INSERM U26, Hôpital Fernand Widal, Paris, France). The RBE4 cell line has been obtained after transfection of a primary rat brain endothelial cell culture with the plasmid pE1A-adenovirus encoding gene [27]. The human cerebral microvascular endothelial cell line hCMEC/D3 was kindly provided by Dr. Pierre-Olivier Couraud (Institute COCHIN, Paris, France). The hTERT/SV40-immortalized hCMEC/D3 clonal cell line was derived from human temporal lobe microvessels isolated from tissue resected during epilepsy surgery [28].

Doxycycline-inducible *MDR1*-EGFP-transduced variants (RBE4-*MDR1*-EGFP, hCMEC/D3-*MDR1-*EGFP) were derived from WT cells by lentiviral transduction as described previously for the hCMEC/D3 cell line, using an *MDR1*-linker-EGFP vector (with EGFP located at the C-terminus of human *MDR1*) kindly provided by Prof. Piet Borst (The Netherlands Cancer Institute, Amsterdam, The Netherlands) as template [31]. The linker sequence in the vector fusing EGFP and *MDR1* consists of 36 bp (TCGACGGTACCGCGGGCCCGGGATCCATCGCCACC). hCMEC/D3-WT, RBE4-WT, and hCMEC/D3-*MDR1*-EGFP cell lines were cultivated as previously described [13, 36]. The same culture conditions were used for the newly prepared RBE4-*MDR1*-EGFP cell line. As described previously [13, 36], hydrocortisone (1.4 µM) was added to the culture medium to enhance the expression and functionality of Pgp and transendothelial resistance [23, 36]. All endothelial cell lines were grown on rat-tail collagen I (60 µg/mL, Gibco) coated 100 mm cultures dishes or permeable membrane supports at 37 °C and 5% CO_2._ Pgp-EGFP protein expression in *MDR1*-EGFP transduced cells was induced by cell cultivation in medium containing 1 µg/µL doxycycline. Expression of Pgp-EGFP was regularly verified by observing the cells with an inverted fluorescence microscope. Doxycycline supplementation of the medium was maintained during the whole culturing period of Pgp-EGFP expressing cells. For experiments, hCMEC/D3 and RBE4 cells were seeded in a density of 50,000 cells/cm^2^. Immortalized *MDR1* transfected porcine kidney LLC-PK1 (LLC-*MDR1*) cells, which were used as a reference standard for vectorial transport experiments, were kindly provided by Prof. Piet Borst. We have used these cells previously for studying vectorial transport of Pgp substrates in the concentration equilibrium transport assay (CETA), which was also used in the present study (see below). Cells were cultivated in medium 199 (Gibco) supplemented with 10% fetal calf serum (FCS, Linaris), and penicillin (100 U/mL)/streptomycin (100 µg/mL) (Merck) and were seeded at a density of 300,000 cells/cm^2^ for experiments. All experiments were carried out between passage 13–35 at 5-days post-confluency (equal to 7 days after seeding).

### Primary cultures of rat brain capillary endothelial cells

The RBE4 and hCMEC/D3 cell lines used here are immortalized, which has the advantage that these cells are stable for several passages and may yield a large number of endothelial cells with the same genetic and phenotypical characteristics. The immortalization status, however, causes several alterations as compared to the native original cell type, including low junctional tightness of cell monolayers and reduced expression of efflux transporters such as Pgp [23]. Therefore, for comparison with the *MDR1*-EGFP transduced rat RBE4 cells, experiments were also performed with primary cultured rat brain capillary endothelial cells (rBCECs). As described by us previously [36], rBCECs were prepared following a protocol by Régina et al. [37] and Perrière et al. [38] from 2 to 3 weeks old Wistar rats (obtained from Charles River; Sulzfeld, Germany). All animals were treated according to protocols evaluated and approved by the ethical committee of our university. In short, meninges were removed from cerebral cortices in phosphate-buffered saline (PBS) with 1% penicillin (100 U/mL)/streptomycin (100 μg/mL) (Biochrom AG) on ice. Subsequently, cortices were mechanically dissociated with scalpels. The homogenate was suspended in enzyme solution: DMEM/F-12 (Gibco®/Life Technologies) supplemented with 1 mg/mL dispase II (Roche), 0.1 mg/mL DNAse I (Roche), 270 U/mL collagenase II (Biochrom AG), and 1% penicillin (100 U/mL)/streptomycin (100 μg/mL) (Biochrom AG) and was incubated for 1.5 h at 37 °C with gentle agitation. The homogenate was centrifuged in 20% bovine serum albumin (Linaris GmbH) solution (1000*g*, 15 min, 4 °C) and the obtained cell pellet was incubated again in the enzyme solution for 1 h at 37 °C. The resulting homogenate was filtered and the retained capillary fragments were removed from the filter with EBM-2 (Lonza) medium supplemented with 20% FCS, 1% penicillin (100 U/mL)/streptomycin (100 μg/mL) (Biochrom), 1 mM HEPES (Gibco®/Life Technologies), 1% l-glutamine (200 mM, Sigma-Aldrich), and 0.5 μg/mL hydrocortisone (Sigma-Aldrich). The cells were seeded on collagen type IV (Sigma-Aldrich) coated 28 cm^2^ plates and cultivated for 72 h in presence of 4 μg/mL puromycin (Sigma-Aldrich) for removal of pericytes [38] in humidified 5% CO_2_/95% air at 37 °C. Thereafter, puromycin was removed and replaced by 2 ng/mL basic FGF (Gibco®/Life Technologies). Cells were cultured until confluency and then seeded for western blot, determination of TEER, Rho123 uptake assay, and vectorial drug transport assay essentially as described in the following for RBE4 and hCMEC/D3 cells.

### Isolation of rat brain capillaries

Capillaries were isolated from the gray matter of four rat brains, according to protocols kindly provided by Drs. Elena Puris (Ruprecht-Karls University, Heidelberg, Germany) and Björn Bauer (University of Kentucky, Lexington, KY, USA). Wistar rats (3 months old) were anesthetized by CO_2_ inhalation and subsequently decapitated. Brains were removed immediately and placed on ice. After removing meninges, choroid plexus, and large superficial blood vessels by rolling each brain on Whatman® blotting paper, cortices were separated from the cerebellum, brain stem, and white matter. Pieces of cortical gray matter were homogenized in 5 volumes (w/v) of buffer A (101 mM NaCl, 4.6 mM KCl, 5 mM CaCl_2_, 1.2 mM KH_2_PO_4_, 1.2 mM MgSO_4_, 15 mM HEPES, 5 mM D-glucose, 1 mM sodium pyruvate, pH 7.4) using a dounce tissue homogenizer. After centrifugation (2000*g*, 10 min, 4 °C) the pellet was resuspended thoroughly in buffer B (buffer A with additional 16% dextran, Sigma-Aldrich) and capillaries were separated from the remaining brain parenchyma by centrifugation in a swinging bucket rotor at 4500*g* for 20 min at 4 °C without brake. The pellet was suspended in 10 mL buffer C (buffer A supplemented with 0.5% BSA) and the resulting suspension was passed sequentially through cell strainers with mesh sizes of 200 µm, 100 µm, and 30 µm. The fraction retained on the 30 µm cell strainer was collected in 40 mL buffer C and centrifuged at 1000*g* for 5 min at 4 °C to pellet capillaries, which were then washed twice with PBS and analyzed by western blotting.

### Western blot analysis

Pgp expression in immortalized cell lines, primary cells, and brain capillaries was evaluated by western blotting. Cells were scraped in radioimmunoprecipitation assay (RIPA) buffer (20 mM Tris, 50 mM NaCl, 0.5% (w/v) sodium deoxycholate, 0.5% (v/v) Triton X-100) supplemented with a protease inhibitor cocktail (Roche) and mechanically lysed by passage through a 21 G syringe needle. Accordingly, brain capillaries were lysed in RIPA buffer. Cell debris was removed by centrifugation and protein concentrations were determined using a BCA protein assay kit (Thermo Fisher Scientific). Equal amounts of protein were loaded onto 10% handcasted SDS-polyacrylamide gels or 4–20% Mini-PROTEAN gels (Bio-Rad) and the separated proteins were then transferred to a PVDF-membrane (Roth). After blocking in 5% milk (Roth) membranes were incubated with anti-Pgp (1:100, Enzo, #ALX-801-002-C100) or anti-β-actin (1:4000, Sigma-Aldrich, #A2066) antibodies for 1 h at room temperature. Secondary antibody incubation was performed for 45 min at room temperature with HRP-conjugated anti-rabbit (1:1000, Dako, #P0448) or anti-mouse (1:1000, Dako, #P0260) antibodies. Protein bands were visualized with the SuperSignal™ West Femto Maximum Sensitivity Substrate (Thermo Fisher Scientific) and detected utilizing the ChemiDoc MP Imager (Bio-Rad). Densitometric protein band quantification was conducted with the Image Lab software version 6.1 (Bio-Rad). Expression of Pgp is reported normalized to β-actin.

### Transendothelial electrical resistance (TEER) measurement

The barrier integrity of hCMEC/D3 and RBE4 cells was monitored using the non-invasive TEER measurement that quantifies the electrical resistance of a cell monolayer. Cells were grown on rat-tail collagen I (60 µg/mL, Gibco) coated permeable filter inserts [ThinCert®; Greiner Bio-One, 12-well, 0.4 µm pore size, polyethylene terephthalate (PET)] and subjected to TEER measurements daily starting on day one post-seeding in at least three replicates. Manual TEER measurements were conducted with the EVOM Volt-Ohm resistance meter (World Precision Instruments) equipped with an EndOhm-12 chamber. TEER values were reported as the measured electrical resistance multiplied by the growth area of the filter inserts and were calculated by subtracting the measured TEER of a coated filter insert without cells from the TEER of a coated filter insert with cells. For comparison of TEER values measured in the ThinCert® system, TEERs were also measured in the Transwell® system from Corning (Corning Costar Corporation, Cambridge, MA) and tissue culture (TC−) inserts from Sarstedt (Nümbrecht, Germany). Furthermore, 6-well and 12-well multiwell plates and different membrane materials were compared (Additional file 1).

### Mannitol permeability assay

The functional tightness of hCMEC/D3-WT, hCMEC/D3-*MDR1*-EGFP, RBE4-WT, and RBE4-*MDR1*-EGFP cell monolayers grown on filter inserts was evaluated by their paracellular permeability to radiolabeled D-mannitol (MW = 182.2 Da, Hartmann Analytic). Seven days post-seeding, the medium in the basolateral donor chamber of the 12-well ThinCert® system was replaced by Opti-MEM (12-well: 1.5 mL, 6-well: 2.7 mL) containing d-[^14^C]mannitol (specific activity 2.035 GBq/mmol) in a concentration of 1.85 kBq/mL. The upper compartment was filled with mannitol-free assay buffer (12-well: 0.7 mL, 6-well: 2 mL) to measure transport in the basolateral-to-apical direction (b-A). Incubation was performed under cell culture conditions (37 °C, 5% CO_2_, humidified atmosphere) on a horizontal shaker (55 rpm). After 30, 60, 120, and 180 min, samples (12-well: 50 µL, 6-well: 130 µL) were collected from the apical chamber and the amount of transported d-mannitol was quantified by liquid scintillation counting using the 1450 Microbeta Trilux liquid scintillation counter (Perkin Elmer Wallac). Basolateral volume was adjusted after each sampling to compensate for changes in the hydrostatic pressure. TEER measurements before and after the assay were carried out to confirm barrier integrity. The apparent permeability coefficients (P_app_) in nm per second were calculated according to Artursson [39]. Each experiment was performed in at least three replicates. As was described for the TEER measurements above, mannitol fluxes were compared in the ThinCert®, Transwell®, and Sarstedt-TC-insert systems, using 6-well and 12-well multiwell plates and different membrane materials. Based on this comparison, all subsequent experiments were performed with ThinCert® cell culture inserts [12-well, 0.4 µm pore size, polyethylene terephthalate (PET)] from Greiner Bio-One.

### Rhodamine 123 uptake/accumulation assay

The functionality of Pgp in RBE4-WT, RBE4-*MDR1*-EGFP, hCMEC/D3-WT, hCMEC/D3-*MDR1*-EGFP cells, and primary rBCECs was analyzed by measuring the uptake of the fluorescent Pgp substrate Rho123 (Sigma-Aldrich). Cells were grown on 6-well plates and cultured for seven days in the presence or absence of doxycycline (1 µg/mL, Biochrom). On the day of the assay, cells were incubated with the Pgp inhibitor tariquidar (0.5 µM, dissolved in DMSO) or the dissolvent in Opti-MEM for one hour under cell culture conditions on a horizontal shaker (55 rpm). Afterward, Rho123 (5 µM, dissolved in ethanol) was added and cells were incubated for another two hours. Rho123 transport was terminated by washing the cells twice with ice-cold PBS. Intracellular Rho123 concentration was determined by cell lysis in standard lysis buffer (25 mM Tris, 50 mM NaCl, 0.5% (w/v) sodium deoxycholate, 0.5% (w/v) Triton X-100) and measurement of fluorescence intensity with the FLUOstar OPTIMA (ex.: 485 nm, em.: 520 nm; BMG Labtech). Protein concentrations were determined using a BCA assay kit (Thermo Fisher Scientific). Rho123 uptake was obtained for each condition in triplicates and calculated as absolute fluorescence per mg protein. Additionally, Pgp functionality was expressed as multidrug resistance activity factor (MAF) as described by Huber et al. [40]. This factor describes Pgp efflux activity based on differences in intracellular Rho123 accumulation in cells after Pgp inhibition compared to cells with active Pgp-mediated efflux. Thus, higher MAF values correlate with increased Pgp activity. For better comparison, differences in Rho123 accumulation are normalized to intracellular Rho123 levels in inhibitor-treated cells. MAF values for Pgp were calculated according to Huber et al. 2012 utilizing the following equation: MAF (%) = 100 ·  (MFI_TQ_ − MFI_0_)/(MFI_TQ_) where MFI_TQ_ and MFI_0_ represent the mean fluorescence intensity of intracellular Rho123 in tariquidar (TQ)-treated and non-treated cells, respectively.

### Vectorial drug transport assay

For these experiments, a concentration equilibrium transport assay (CETA) was used, which is more sensitive to identify Pgp substrates than conventional bidirectional (concentration gradient) transport assays [41]. In the CETA, the drug is added to both (apical and basolateral) sides of the monolayer, so that the initial drug concentration is the same in both compartments, thereby minimizing the effect of passive diffusion across the cell monolayer. Particularly for lipophilic compounds, passive transcellular diffusion could form a bias in Transwell assays by concealing active transport, which we have previously demonstrated by comparing vectorial drug transport of various small lipophilic drugs in conventional bidirectional assays versus drug transport in CETA [41].

In the present study, the CETA assay was used to determine the flux of the radiolabeled Pgp substrate [*N*-methyl-^3^H]N-desmethyl loperamide ([^3^H]dLop) across hCMEC/D3-*MDR1*-EGFP and RBE4-*MDR1*-EGFP monolayers. Although Kannan et al. [42] have shown that [^3^H]dLop is trapped in lysosomes, this does not restrict its use for studying Pgp-mediated vectorial drug transport in BCECs, as demonstrated recently by us in primary cultured porcine BCECs [43]. Cells were grown on permeable ThinCert® filter supports (12-well, 0.4 µm pore size, PET, Greiner Bio-One). LLC-*MDR1* monolayers were used as a reference standard for the demonstration of vectorial transport of the Pgp substrate ([^3^H]dLop) used in these experiments. Transport studies were performed in triplicates seven days after seeding. Before substrate exposure, the culture medium was replaced by Opti-MEM (Gibco) with or without the Pgp inhibitor verapamil (20 µM) for 1 h in the apical and basolateral chambers. Verapamil was preferred to tariquidar (which was used in the Rho123 accumulation experiments) because tariquidar has been shown to compete for lysosomal trapping of [^3^H]dLop [42], which may form a bias for data on vectorial drug transport at the cell membrane. To initiate transport studies, [^3^H]dLop was added to the upper and lower compartment in a concentration of 5 nM in the absence or presence of verapamil (20 µM). After 30, 60, 120, 180, and 240 min, samples of 50 µL and 70 µL were retrieved from the apical and basolateral chambers, respectively. Quantification of substrate transport was conducted by liquid scintillation counting using a 1450 Microbeta Trilux liquid scintillation counter (Perkin Elmer Wallac). All incubation steps were performed under cell culture conditions on a horizontal shaker (55 rpm) with 0.7 mL medium in the upper and 1.5 mL medium in the lower compartment. Barrier tightness was confirmed via TEER measurements before and after the assay. Baseline dLop levels were corrected by the amount of dLop absorbed by the filter system as measured in blank inserts without cells.

Additionally, vectorial drug transport across RBE4-*MDR1*-EGFP, hCMEC/D3-*MDR1-EGFP*, LLC-*MDR1*, and primary rBCEC monolayers was studied using 2 µM Rho123 as a fluorescent Pgp substrate essentially following the same protocol as described for [^3^H]dLop. Transport was measured using a FLUOstar OPTIMA fluorescence reader (ex.: 485 nm, em.: 520 nm; BMG Labtech), analyzing samples from basolateral and apical compartments collected at the various time points.

### Microscopy

Cell morphology of hCMEC/D3, RBE4, rBCECs, and LLC cells was assessed via phase-contrast microscopy. Therefore, cells were seeded onto 6-well cell culture dishes and analyzed after confluence with an inverted fluorescence microscope (Olympus IX-70, Hamburg, Germany) and a 10× objective.

Pgp localization was visualized in confluent RBE4- and hCMEC/D3-*MDR1*-EGFP monocultures grown on glass coverslips by confocal fluorescence microscopy and live-cell imaging.

Intercellular Pgp transfer was studied by coculturing RBE4-WT and –*MDR1*-EGFP cells to equal amounts (50:50) on glass coverslips until confluency. Wildtype cells were prelabeled with either Cell Proliferation Dye eFluor 670 (5 µM, 10 min, 37 °C, eBioscience) or CellTracker red CMTPX (5 µM, 25 min, 37 °C, Thermo Fisher Scientific) for identification in the monolayer after coculturing. Since Pgp-EGFP is exclusively expressed by *MDR1*-EGFP transduced cells and not by WT cells (which exhibit only low intrinsic Pgp levels), Pgp transfer between transduced and WT cells can be easily traced by the green fluorescent signal of the EGFP-tagged Pgp. Cell nuclei were stained with bisbenzimide H (5 mM, 5 min, 37 °C, Sigma-Aldrich). Images were acquired by confocal fluorescence microscopy and live-cell imaging.

Intracellular distribution of the autofluorescent Pgp substrate doxorubicin was investigated in cocultures of RBE4-WT and -*MDR1*-EGFP cells seeded on glass coverslips until confluency. Coculturing of WT and *MDR1*-EGFP cells allowed to compare the differential intracellular distribution of the autofluorescent Pgp substrate in WT cells with low intrinsic expression of Pgp and *MDR1*-EGFP transduced cells with a higher Pgp expression. Cells were incubated with LysoTracker (75 nM, 1 h, 37 °C, Thermo Fisher Scientific) and doxorubicin (10 µM, 30 min, 37 °C, Enzo) before live-cell imaging and confocal microscopy. For imaging, glass coverslips were mounted into a PeCon open chamber (PeCon, Erbach, Germany), covered with serum-free Opti-MEM, and kept at 37 °C. Endolysosomal staining in *MDR1*-EGFP transduced RBE4 and hCMEC/D3 cell cultures was performed similarly, however, without doxorubicin incubation.

Confocal fluorescence microscopy was conducted with a Leica TCS SP5 II fluorescence microscope (Leica Microsystems, Bensheim, Germany) combined with a 63 × 1.2 water immersion objective. For excitation, wavelengths of 405 nm (bisbenzimide H, LysoTracker), 561 nm (doxorubicin, CMTPX), 633 nm (eFluor 670), and 488 nm (Pgp-EGFP) were used.

For analysis of the effects of doxorubicin on Pgp-inhibited cells, confluent monolayers of RBE4- and hCMEC/D3-*MDR1*-EGFP cells grown on coverslips were preincubated with or without the Pgp inhibitor tariquidar (0.5 µM, 1 h, 37 °C) in Opti-MEM before the addition of doxorubicin (10 µM, 30 min, 37 °C). After substrate incubation, cells were covered with cell culture medium for 24 h. Cells were then washed once with PBS, fixed with paraformaldehyde (4%, 30 min), and permeabilized with Triton X-100 (1%, 5 min). Finally, cells were washed and coverslips were mounted in Prolong Gold antifade (Carl Roth GmbH, Karlsruhe, Germany) containing 4’,6-diamidino-2-phenylindole (DAPI) to stain nuclei. Analysis was performed with a Zeiss Axio Observer fluorescence microscope including an ApoTome.2 and using a 40× or 63× objective.

To evaluate the number of green-fluorescent (Pgp-EGFP positive) intracellular vesicles in RBE4- and hCMEC/D3-*MDR1*-EGFP cells before and 24 h after treatment with doxorubicin (10 µM, 30 min, 37 °C), imaging of the cells was performed using an inverted fluorescence microscope Lumascope 620 (Etaluma Carlsbach, USA) placed in the cell culture incubator. After treatment with doxorubicin, cells were maintained in a doxorubicin-free cell culture medium until imaging. Before imaging, cellular debris was removed by washing the cells twice with sterile PBS followed by the addition of fresh doxorubicin-free culture medium.

Following fixation of the cells with paraformaldehyde or ice-cold methanol (10 min, − 20 °C) indirect Pgp staining was performed as described above using a primary monoclonal antibody against Pgp (1:100, Sigma-Aldrich, #P7965) and a secondary Alexa Fluor 568 antibody (1:500, Thermo Fisher Scientific, #A11004).

### Statistics

Data are presented as means ± standard error of the mean (SEM) of at least three biological replicates. An unpaired t-test was applied to assess the significance of intergroup differences. Significant differences between multiple groups within one experiment were either calculated by one-way or two-way analysis of variance (ANOVA) followed by Dunnett’s or Bonferroni post hoc tests. P values < 0.05 were considered statistically significant. Statistical analyses were conducted using the PRISM 8 software (GraphPad Software Inc.).

## Results

### Morphology of wildtype and *MDR1*-EGFP transduced RBE4 and hCMEC/D3 cells and localization of Pgp-EGFP

As shown in Additional file 2 (A, B), both hCMEC/D3-WT cells and RBE4-WT cells formed monolayers and exhibited elongated spindle-shaped morphology when examined at confluence by phase-contrast microscopy, substantiating previous reports [13, 36]. The transduction with *MDR1*-EGFP did not alter the morphology of the cells (Additional file 2 (C, D)). As shown in Additional file 3A, primary cultured rat BCECs (rBCECs) exhibited similar morphology to that observed for the immortalized BCEC lines. Additional file 3B illustrates the morphology of the LLC-*MDR1* kidney epithelial cell monolayers used for comparison with BCECs here (see below).

**Fig. 1:**
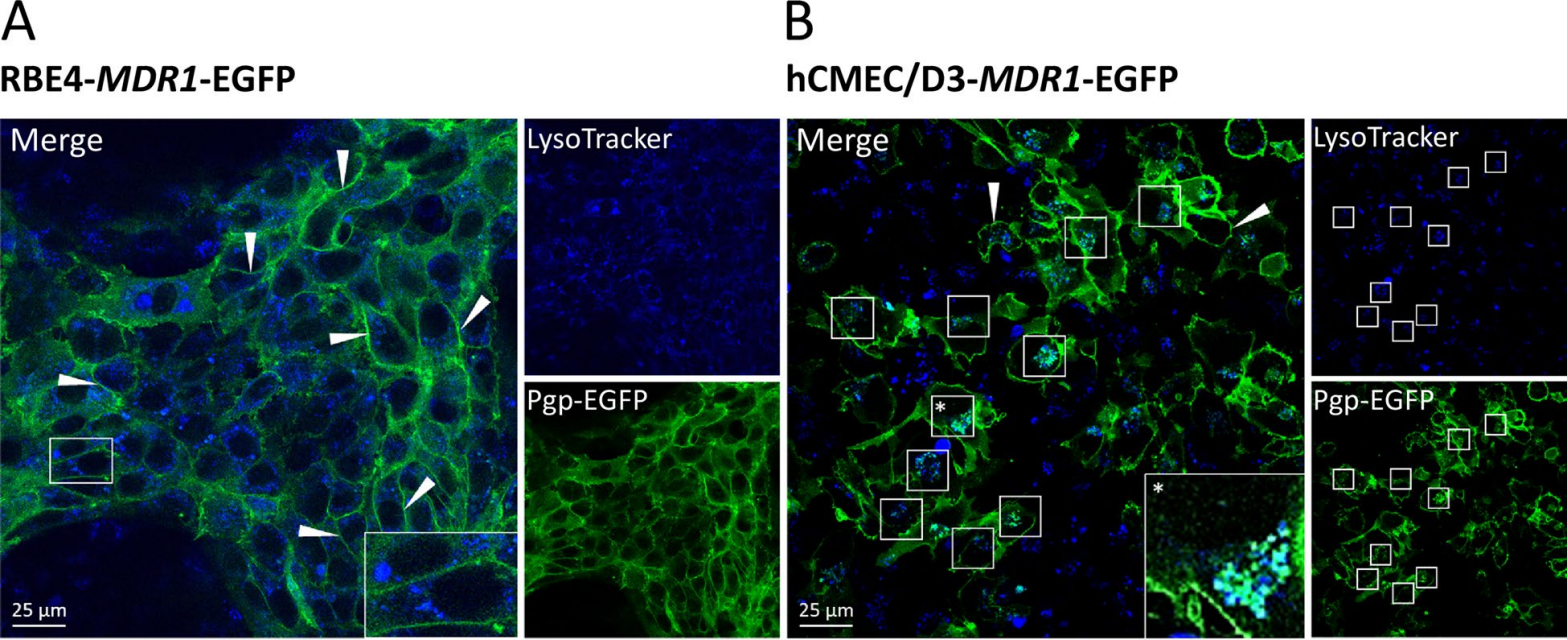
Subcellular localization of Pgp in RBE4- and hCMEC/D3-*MDR1*-EGFP cultures and endolysosomal staining. *MDR1*-EGFP transduced RBE4 (**A**) and hCMEC/D3 (**B**) cells grown on glass coverslips were treated with the fluorescent acidotropic LysoTracker probe to stain endolysosomal vesicles. Pgp localization visualized by green fluorescence of the EGFP protein tag and colocalization with endolysosomal vesicles was assessed by live-cell imaging and confocal laser scanning microscopy. Imaging revealed that, as expected, Pgp-EGFP (green) was localized at the cell surface in both cell lines (arrows). In hCMEC/D3-*MDR1*-EGFP cells, Pgp-EGFP (green) colocalized with LysoTracker (blue) stained intracellular vesicles (boxes in **B**), whereas this was hardly observed in RBE4-*MDR1*-EGFP cells. Boxes in the bottom right corner of the merged images show magnifications of Pgp subcellular localization in the respective cell line

However, differences were observed in the localization of Pgp-EGFP in the two immortalized BCEC lines when using confocal laser scanning fluorescence microscopy and live-cell imaging (Additional file 4). In RBE4 cells, Pgp-EGFP was predominantly expressed at the cell surface (Additional file 4A), consistent with its function as an efflux transporter, whereas, as reported previously [13, 31], in hCMEC/D3 cells, the Pgp-EGFP fusion protein was visible both at the plasma membrane and in intracellular vesicles, most likely presenting endolysosomal compartments (Additional file 4B). To demonstrate that the Pgp-EGFP-positive intracellular vesicles are of endolysosomal origin, we co-stained *MDR1*-EGFP-transduced RBE4 and hCMEC/D3 cells with a endolysosomal marker (LysoTracker). As shown in Fig. 1B, endolysosomal vesicles were positive for Pgp-EGFP fluorescence signal in hCMEC/D3 cells, whereas intracellular Pgp-EGFP localization was hardly observed in RBE4 cells (Fig. 1A). These results further substantiated findings from our previous studies with endolysosomal markers in hCMEC/D3-*MDR1*-EGFP cell cultures that the Pgp-EGFP-positive intracellular vesicles are of endolysosomal origin [13, 31]. The marked difference in numbers of Pgp-EGFP-positive intracellular vesicles in RBE4 versus hCMEC/D3 cells was confirmed by counting these vesicles in both cell lines (Additional file 5).

To prove that in cells expressing the Pgp-EGFP fusion protein, EGFP is not simply cleaved and being sequestered in intracellular vesicles, we evaluated whether an antibody to Pgp colocalizes to the EGFP signal. As shown in Additional file 6, indirect staining of Pgp in *MDR1*-EGFP transduced RBE4 and hCMEC/D3 cells colocalized with the Pgp-EGFP fluorescence signal in intracellular vesicles (in hCMEC/D3) and in the cell membrane of both RBE4 and hCMEC/D3 cells.

Furthermore, we also stained Pgp in RBE4-WT cells, showing that Pgp was exclusively expressed at the cell surface in these cells (Additional file 7). In contrast, Pgp protein expression in hCMEC/D3-WT cells has been described both in the plasma membrane and in intracellular vesicles [44, 45], substantiating the present findings in *MDR1*-EGFP-transduced hCMEC/D3 cells (Fig. 1B). This precludes the possibility that intracellular localization of Pgp observed in *MDR1*-EGFP-transduced hCMEC/D3 cells was a result of the EGFP tag. If that were the case, intracellular Pgp localization should have also been observed in the *MDR1*-EGFP-transduced RBE4 cells, which it was not. Moreover, the demonstration of vesicular localization of Pgp in different studies on hCMEC/D3 cells [13, 31, 32, 44, 45] argues against the possibility that the differences between hCMEC/D3 and RBE4 cells reported here were simply due to transfected or immortalized clone differences.

### Expression of Pgp in wildtype and *MDR1*-EGFP transduced RBE4 and hCMEC/D3 cells

**Fig. 2:**
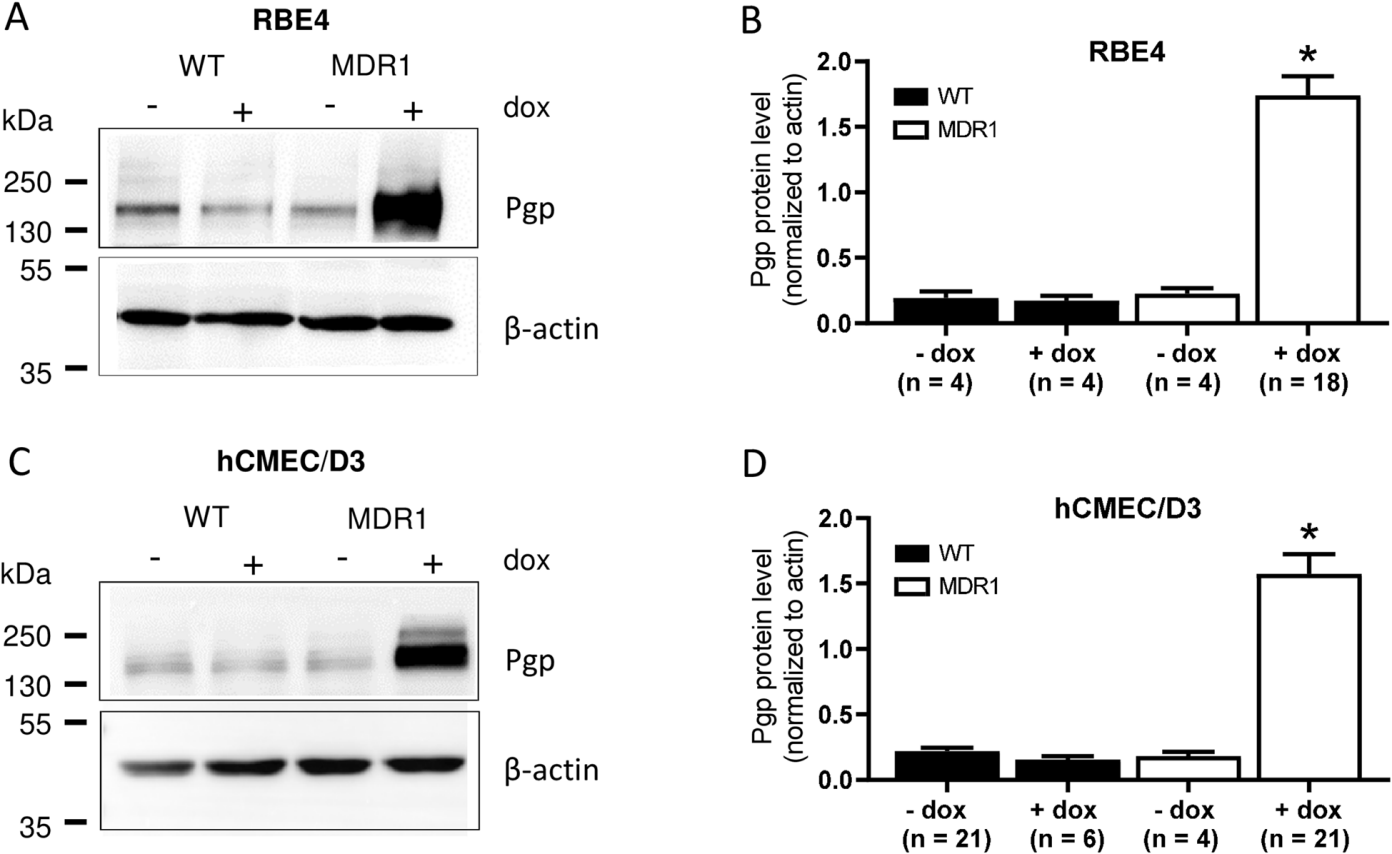
P-glycoprotein expression in hCMEC/D3 and RBE4 wildtype and *MDR1*-EGFP transduced cells. Representative western blots (30 µg protein/sample) (**A**, **C**) and quantification (**B**, **D**) of Pgp expression in the different cell lines. In both hCMEC/D3- and RBE4-*MDR1*-EGFP cells, the addition of doxycycline (dox) leads to the induction of Pgp expression. In comparison to wild-type (WT) cells, Pgp expression in RBE4- and hCMEC/D3-*MDR1*-EGFP cells in the presence of dox was increased by 9- and sevenfold, respectively. Dox did not affect Pgp expression in WT cells. Data are presented as means + SEM; sample size (biological replicates) is shown below the bars. Asterisks indicate significant differences between cell types as calculated by one-way ANOVA followed by Dunnett’s multiple comparisons test (P < 0.0001)

**Fig. 3:**
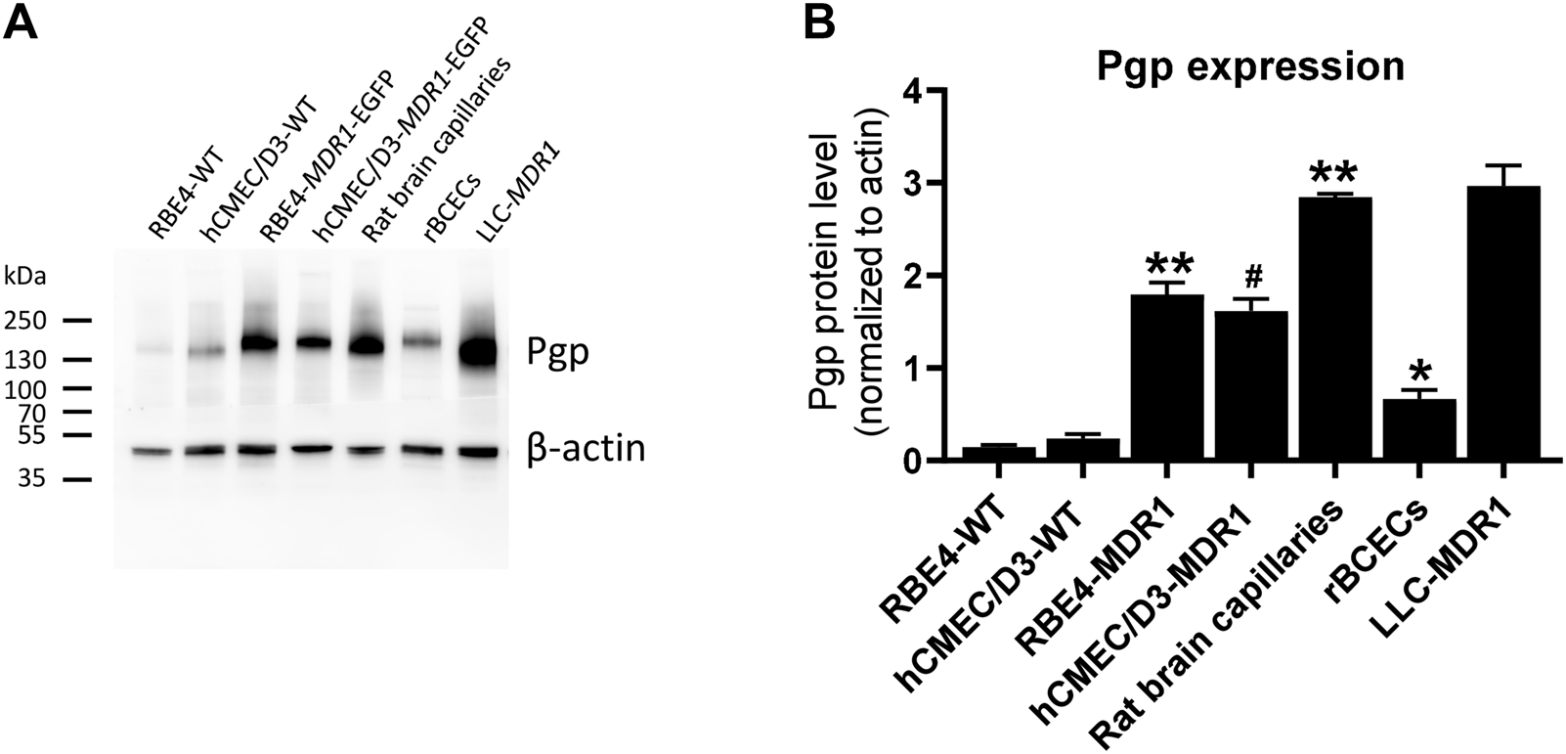
P-glycoprotein expression in hCMEC/D3 and RBE4 wildtype and *MDR1*-EGFP transduced cells in comparison to freshly prepared rat brain capillaries, primary cultured rat brain capillary endothelial cells, and *MDR1*-transfected LLC cells. Representative western blots (12 µg protein/sample) (**A**) and quantification (**B**) of Pgp expression. Pgp content of the immortalized BCEC lines (RBE4, hCMEC/D3) was determined in the presence of doxycycline. Data are presented as means + SEM; sample size (biological replicates) was 8 (RBE4-WT), 10 (hCMEC/D3-WT), 21 (RBE4-MDR1), 25 (hCMEC/D3-MDR1), 2 (rat brain capillaries), 6 (primary rBCECs), and 5 (LLC-MDR1), respectively. Data were analyzed by one-way ANOVA followed by Dunnett’s multiple comparisons test. Asterisks indicate significant differences of RBE4-MDR1, rat brain capillaries, and rBCECs to RBE4-WT (*P < 0.05; **P < 0.0001), whereas the hash sign indicated a significant difference between hCMEC/D3-MDR1 vs. hCMEC/D3-WT (^#^P < 0.0001)

As shown in Fig. 2, RBE4-WT and hCMEC/D3-WT cells exhibited approximately the same low expression of Pgp, which was not affected by doxycycline. A similar low Pgp expression was observed in *MDR1*-EGFP transduced cells in the absence of doxycycline. In the presence of doxycycline, Pgp expression increased seven to ninefold in *MDR1*-EGFP transduced cells when compared to WT controls (Fig. 2B, D). The Pgp band in the induced cells was too intense to allow separating endogenous Pgp (170 kDa) and Pgp-EGFP (200 kDa). However, when a lower amount of protein was used for western blot analysis, the shift in the Pgp-EGFP fusion bands was visible (Fig. 3A).

For comparison, we determined the Pgp protein expression of primary rBCECs and freshly isolated rat brain capillaries (Fig. 3). The Pgp content of rBCECs was only about 4.5-fold higher than Pgp protein expression in RBE4-WT cells, whereas the Pgp content of freshly isolated rat brain capillaries exceeded the Pgp content of the *MDR1*-EGFP transduced RBE4 cells (Fig. 3B). The about 4-times higher Pgp content of freshly isolated rat brain capillaries vs. primary cultured rBCECs shown in Fig. 3 is in line with previous experiments by Régina et al. [46], who reported that the amount of Pgp was about 4-times higher in isolated rat brain microvessels than in rBCECs prepared from these microvessels. Importantly, the data shown in Fig. 3 demonstrate that transduction of RBE4 cells with *MDR1*-EGFP does not lead to a gross overexpression of the transporter but to intermediate Pgp levels between those of rBCECs and freshly isolated rat brain capillaries. Interestingly, the Pgp content of *MDR1*-transfected LLC cells was similar to that of freshly isolated rat brain capillaries (Fig. 3B).

### Junctional tightness and paracellular permeability of wildtype and *MDR1*-EGFP transduced RBE4 and hCMEC/D3 cells

**Fig. 4:**
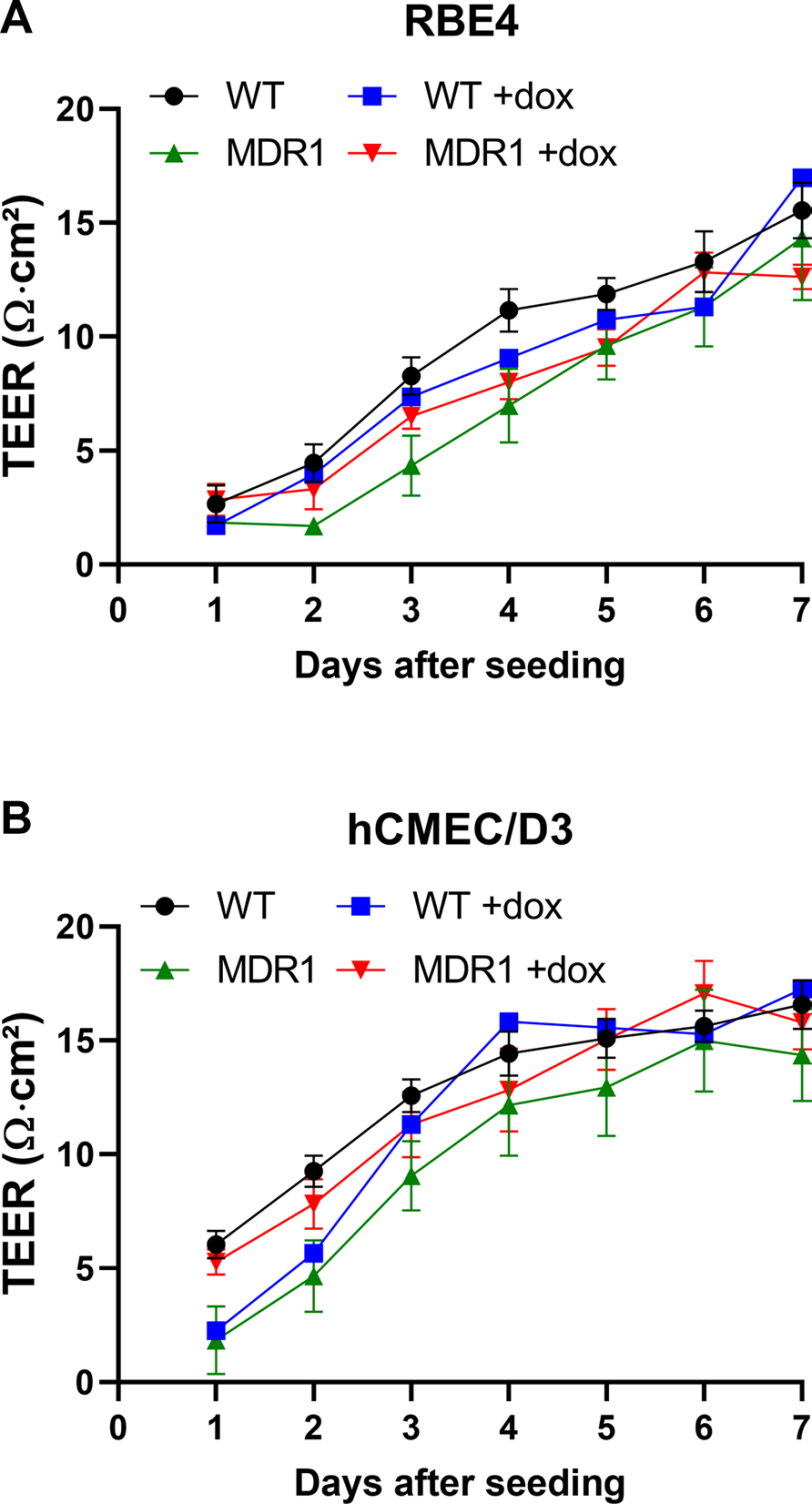
Barrier integrity of monolayers of hCMEC/D3 and RBE4 wildtype and *MDR1*-EGFP expressing cells. Cells were grown on ThinCert® permeable membrane supports and barrier integrity was compared by measurement of transendothelial electrical resistance (TEER). TEER was measured daily over seven days after seeding using an EndOhm chamber. TEER values of 3–8 replicates are presented as means ± SEM. Statistical analysis performed by two-way ANOVA and Bonferroni post hoc test revealed no significant differences of TEER values between cell types

**Fig. 5:**
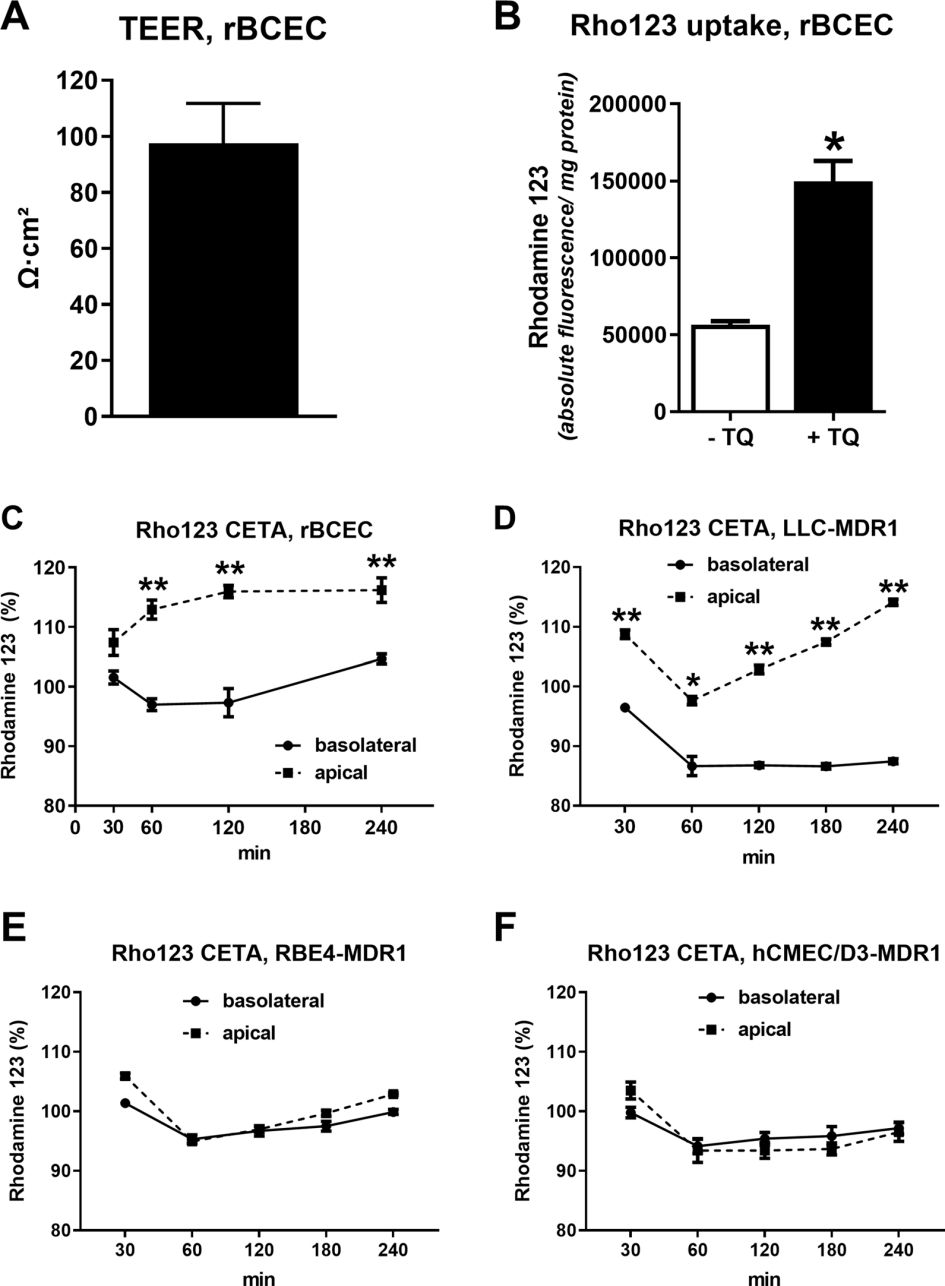
Characteristics of primary cultured rat brain capillary endothelial cells (rBCECs), which were prepared for comparison with the RBE4 rat BCEC line. Pgp expression of these cells is shown in Fig. 3. Average TEER of rBCEC monolayers (**A**), shown as mean ± SEM of four replicates. Rhodamine 123 (Rho123) uptake assay in the absence (− TQ) or presence of the Pgp inhibitor tariquidar (0.5 µM, + TQ) (**B**). Data are shown as mean ± SEM of six replicates. Student’s t test revealed a significant effect of tariquidar as indicated by an asterisk (P < 0.0001). Directional Pgp transport assay (CETA), using Rho123 as a Pgp substrate (**C**–**F**). Data are shown as mean ± SEM of four replicates. Data were analyzed by two-way ANOVA followed by Bonferroni post hoc tests. Rho123 is transported from the basolateral to the apical chamber, leading to significant concentration differences between the two chambers (**P < 0.01). The increase in Rho123 levels in the basolateral chamber at 240 min could indicate redistribution of this Pgp substrate from the apical to the basolateral chamber by passive diffusion. The average TEER of the cell monolayers used in this assay was 163 Ω cm^2^. For comparison with vectorial drug transport in rBCECs (**C**), Rho123 transport in the CETA is shown for *MDR1* transfected LLC cells (**D**), *MDR1*-EGFP transduced RBE4 cells (**E**), and *MDR1*-EGFP transduced hCMEC/D3 cells (**F**). Data are shown as mean ± SEM of three replicates; data were analyzed by two-way ANOVA followed by Bonferroni post hoc tests; significant differences between drug levels in the apical vs. basolateral chambers are indicated by asterisks (*P < 0.05; **P < 0.01)

As would be expected, transduction of RBE4 and hCMEC/D3 cells with *MDR1*-EGFP did not affect TEER or paracellular mannitol flux of the cells (Fig. 4, Table 1). As shown in Fig. 4A, TEER progressively increased over 7 days after seeding in RBE4 cells, whereas a plateau in TEER values was reached after 4 days in hCMEC/D3 cells. TEER values were comparable in all cell lines, reaching maximal values of ~ 13–16 Ω cm^2^ when measured with an EndOhm chamber. For hCMEC/D3 cells, these low TEER values were comparable to values reported in the literature when using an epithelial Volt-Ohm meter (EVOM) coupled to an EndOhm chamber for TEER measurements [43, 47], whereas TEER measurements with chopstick electrodes result in significantly higher values [21, 43, 48]. When TEER was measured with an EndOhm chamber in primary rBCECs, values were in the range of 80–140 Ω cm^2^ (Fig. 5A), i.e., about 5–10-times higher than the TEER values determined in RBE4 cells. These TEER values of rBCECs were similar to the values (123 ± 1.1 Ω cm^2^) reported previously for these cells [38].

**Table 1:** TEER values and paracellular mannitol permeability (basolateral to apical) in nm/s and percentage of transport per hour of RBE4 and hCMEC/D3 cells were measured 7 days after seeding

Cell type	TEER	Mannitol permeability	
	(Ω cm^2^)	(nm/s)	(%/h)
RBE4-WT	15.55 ± 1.22	59.58 ± 4.07	4.20 ± 0.30
RBE4-*MDR1* + dox	12.63 ± 0.54	60.69 ± 1.59	4.30 ± 0.22*
hCMEC/D3-WT	16.57 ± 1.06	53.76 ± 4.32	3.71 ± 0.13
hCMEC/D3-*MDR1* + dox	15.79 ± 1.18	56.65 ± 2.52	3.81 ± 0.11

Data are presented as the mean ± SEM of 3–8 replicates. Experiments were performed with ThinCert® cell culture inserts (12-well, 0.4 µm pore size, PET membranes). See “Materials and methods” and Additional file 1 for further details. Significant differences as calculated by one-way ANOVA (Bonferroni post hoc test) compared to hCMEC/D3-WT are marked by asterisk. All other values were not significantly different (P < 0.05)

As a consequence of the low TEER values of RBE4 and hCMEC/D3 cells, paracellular permeability (indicating paracellular leakage through endothelial tight junctions) was relatively high with average mannitol flux values of between 54 and 60 nm/sec (Table 1). Mannitol permeability was comparable in RBE4 and hCMEC/D3 cells and was not affected by transduction with *MDR1*-EGFP.

As reported previously for hCMEC/D3 cells [47] and primary murine BCECs [49], the type of tissue culture inserts and membrane materials markedly affected TEER and mannitol flux values of hCMEC/D3 and RBE4 cells as shown in Additional file 1. The highest TEER and lowest mannitol flux data were obtained with ThinCert® 12-well multiwell plates, using PET membranes with a pore density of 2 × 10^6^ and pore size of 0.4 µm (Additional file 1), providing the best growth conditions for the cells to form a tight monolayer. This system was therefore chosen for the data shown in Table 1 and Fig. 4, as well as for all subsequent experiments.

### Pgp is functional in wildtype and *MDR1*-EGFP transduced RBE4 and hCMEC/D3 cells when using a rhodamine 123 uptake assay

**Fig. 6:**
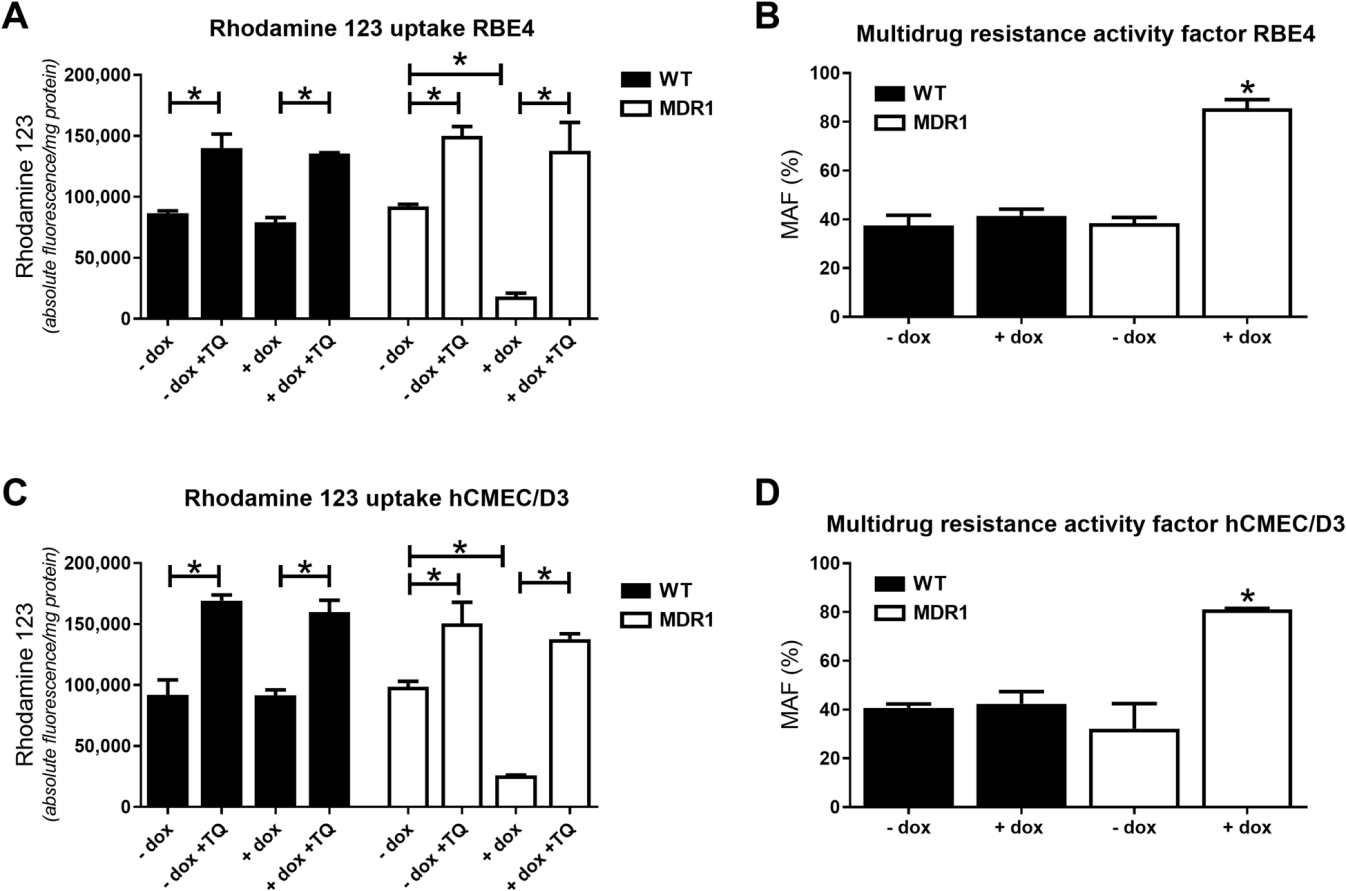
P-glycoprotein transport function in hCMEC/D3 and RBE4 wildtype and *MDR1*-EGFP expressing cells by rhodamine 123 uptake assay. Cells were cultured in 6-well plates with or without doxycycline (dox) and intracellular accumulation of rhodamine 123 (Rho123) was assessed and reported as absolute fluorescence per mg protein in the presence or absence of the Pgp inhibitor tariquidar (**A**, **C**). Intracellular Rho123 accumulation negatively correlates with Pgp activity. Data are shown as means + SEM of three experiments; significant differences in intracellular Rho123 accumulation between cell types are marked by asterisks (P < 0.05). In **B** and **D**, the Pgp function was expressed by calculation of the multidrug resistance activity factor (MAF) according to Huber et al. ([40]; see Methods) as a quantitative measure of multidrug resistance. MAF values are presented as means + SEM of 3 replicates; statistical analysis was performed by one-way ANOVA followed by Bonferroni’s multiple comparisons test; significant differences compared to wildtype control cells (-dox) are indicated by asterisks (P < 0.05)

As shown in Fig. 6A, C, Pgp was functional in RBE4 and hCMEC/D3-WT cells, since the accumulation of Rho123 in the cells was significantly increased by the Pgp inhibitor tariquidar. Doxycycline did not alter the functionality of Pgp in the WT cells. In *MDR1*-EGFP transduced RBE4 cells, in the absence of doxycycline, Rho123 accumulation was similar to that determined in WT cells. In the presence of doxycycline, however, Rho123 accumulation was significantly reduced, indicating increased Pgp-mediated efflux, which could be inhibited by tariquidar (Fig. 6A). When comparing Rho123 accumulation in the absence and presence of tariquidar, Rho123 efflux was increased ~ 7-fold. The increased functionality of Pgp in doxycycline-induced RBE4-*MDR1*-EGFP cells is also demonstrated by calculating the multidrug resistance activity factor (MAF) according to Huber et al. [40] (Fig. 6B). Very similar findings were obtained with hCMEC/D3-*MDR1*-EGFP cells (Fig. 6C, D).

In primary rBCECs, intracellular Rho123 accumulation was significantly lower (Fig. 5B) than Rho123 accumulation in RBE4-WT cells (56,482 ± 2456 vs. 86,513 ± 2021; P = 0.0001), which is in line with the higher Pgp expression of the rBCECs (Fig. 3). Following Pgp inhibition by tariquidar, Rho123 accumulation was similar in rBCECs and RBE4 cells. MAF values calculated for intracellular Rho123 accumulation in rBCECs were significantly higher than MAF values in RBE4-WT cells (61.2% ± 2.7 vs. 37.5% ± 4.2; P = 0.002), again in line with the higher Pgp expression of the rBCECs.

### Lack of vectorial drug transport in *MDR1*-EGFP transduced RBE4 and hCMEC/D3 cells

**Fig. 7:**
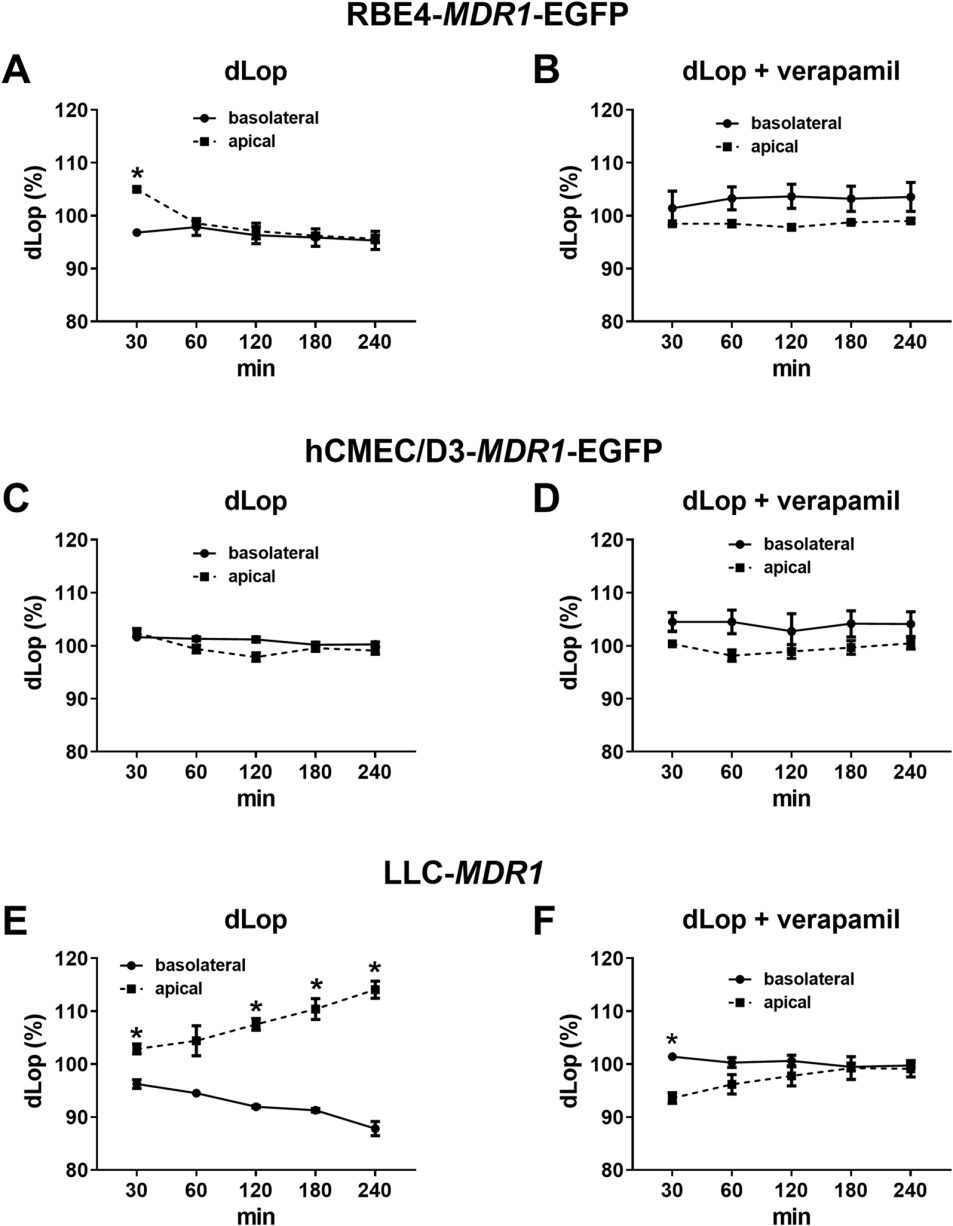
Directional P-glycoprotein transport assay in *MDR1*-EGFP expressing RBE4 and hCMEC/D3 cells. RBE4-*MDR1*-EGFP (**A**, **B**) and hCMEC/D3-*MDR1*-EGFP (**C**, **D**) were grown in the apical compartment of 12-well ThinCert® cell culture inserts, as well as *MDR1*-transfected LLC cells (**E**, **F**) for comparison. For measurement of directional Pgp-mediated transport by the concentration equilibrium transport assay (CETA), equal concentrations of the Pgp substrate [^3^H]dLop were added to the apical and basolateral compartment in the absence (**A**, **C**, **E**) or presence (**B**, **D**, **F**) of the Pgp inhibitor verapamil. Amounts of [^3^H]dLop in the apical and basolateral chamber were measured at various time points and presented as percent of initial drug concentration as mean ± SEM of 3 replicates. Data were analyzed by two-way (ANOVA) followed by Bonferroni post hoc tests. Asterisks show significant differences between the two chambers (P < 0.05)

As shown in Fig. 7A–D, asymmetrical (basolateral to apical) transport of the selective Pgp substrate dLop [50] was not observed in *MDR1*-EGFP transduced RBE4 and hCMEC/D3 cells. In contrast, such transport was obtained in *MDR1* transfected LLC cells (which are widely used as a surrogate Pgp screening model of the BBB [25, 26]), and this transport was almost completely inhibited by verapamil (Fig. 7E, F). TEER of the LLC-*MDR1* cells was 132 Ω cm^2^, i.e., considerably higher than the TEER values of the *MDR1*-EGFP transduced RBE4 and hCMEC/D3 cells (Table 1). Consistent with the high junctional tightness of the LLC monolayers, paracellular mannitol flux was less than 1% of mannitol diffusion per hour (corresponding to a mannitol permeability lower than 12 nm/s) and thus much lower than the mannitol permeability observed in the immortalized BCEC lines (Table 1), which explains that vectorial (Pgp-mediated) drug transport from the basolateral to the apical Transwell chamber was observed in the MDR1-transfected LLC cells but not in the BCEC lines.

In primary rBCECs, we used the Pgp substrate Rho123 to demonstrate vectorial transport. As shown in Fig. 5C, concentrations of Rho123 were significantly higher in the apical vs. basolateral chambers in the CETA assay, indicating asymmetric transport from the basolateral to the apical side. Rho123 was also used for transport (CETA) assays in LLC-*MDR1*, RBE4-*MDR1*-EGFP, and hCMEC/D3-*MDR1*-EGFP cells. Similar to the data with dLop (Fig. 7), significant vectorial transport was observed in LLC-MDR1 cells (Fig. 5D) but not in *MDR1*-EGFP transduced RBE4 and hCMEC/D3 cells (Fig. 5E, F).

### Intracellular distribution of doxorubicin in RBE4 wildtype and -MDR1-EGFP cocultures

**Fig. 8:**
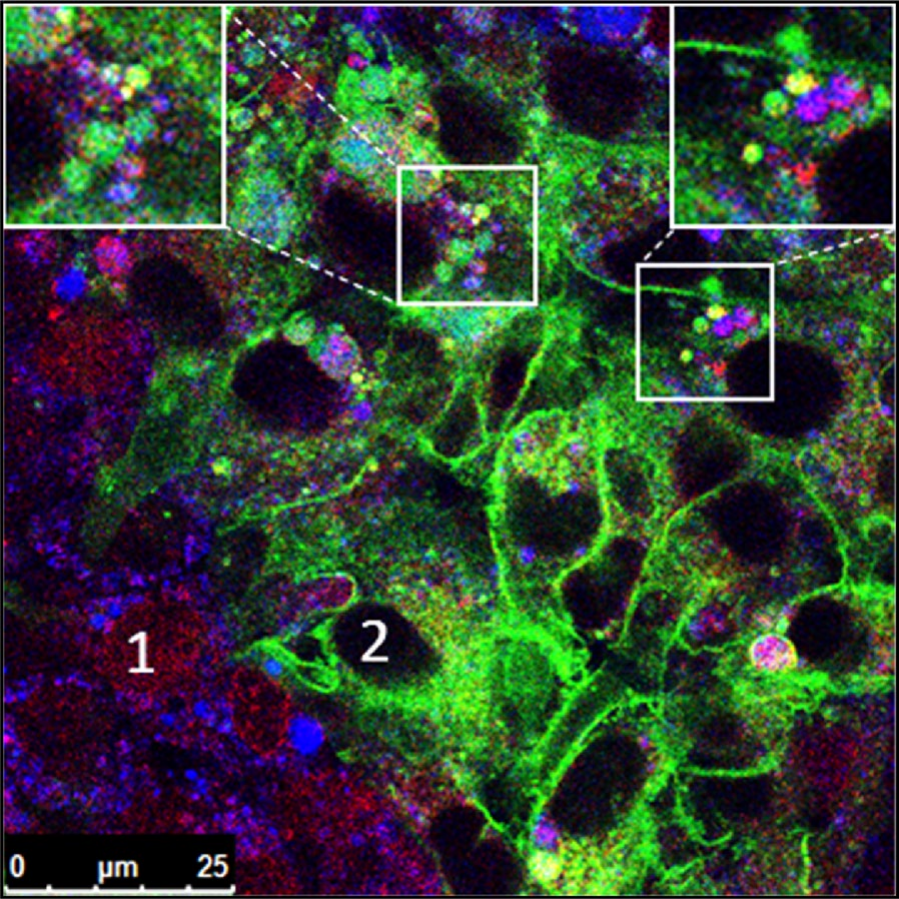
Intracellular distribution of doxorubicin in RBE4 wildtype and -*MDR1*-EGFP cocultures. RBE4 wildtype and –*MDR1*-EGFP cocultures were stained for endolysosomal vesicles with LysoTracker (blue) and treated with the Pgp substrate doxorubicin (red) for 30 min, before live-cell imaging using a confocal laser scanning microscope. Insets show a magnification of endolysosomal localization of doxorubicin and Pgp (green). In RBE4 wildtype cells, doxorubicin binds to its nuclear target as visible by red staining (#1), while nuclei of Pgp-EGFP overexpressing cells lack a nuclear doxorubicin staining (#2). Similar experiments with similar outcomes were previously performed with cocultures of wildtype and *MDR1*-EGFP-transduced hCMEC/D3 cells [31], and therefore these experiments were not repeated and again illustrated here

In addition to actively transporting drugs out of the cell, Pgp has been proposed to mediate lysosomal sequestration of chemotherapeutic drugs in cancer cells, thus contributing to drug resistance [10, 11, 51]. We have recently described that Pgp-mediated lysosomal sequestration of Pgp substrates, including doxorubicin, also occurs in hCMEC/D3 cells and primary cultured porcine BCECs [13]. This prompted us to evaluate whether Pgp-mediated lysosomal trapping of doxorubicin also occurs in RBE4 cells, which, as described above, exhibit markedly less Pgp-EGFP in intracellular compartments compared to hCMEC/D3 cells. For this purpose, the intracellular distribution of doxorubicin was studied in cocultures of RBE4-WT cells with low intrinsic levels of Pgp and Pgp-EGFP overexpressing RBE4-*MDR1*-EGFP cells. Cocultures were exposed to the autofluorescent Pgp substrate doxorubicin after labeling of endolysosomal vesicles with the vital fluorescence dye LysoTracker blue (Fig. 8). Coculturing of WT and *MDR1*-EGFP cells allowed to follow the differential intracellular distribution of doxorubicin in cells with different Pgp protein expression levels. The localization and fate of Pgp in cocultures were easy to trace by the EGFP-tag of Pgp expressed in the RBE4-*MDR1* cells by confocal fluorescence microscopy and live-cell imaging.

Doxorubicin is one of the most widely used clinical anticancer agents and a hydrophobic weak base that intercalates as a primary target into nuclear DNA [52]. Consequently, when RBE4-WT cells (which did not overexpress Pgp) were exposed to doxorubicin, the doxorubicin bound to cell nuclei.

In contrast, this was not seen in Pgp-overexpressing RBE4-*MDR1*-EGFP cells (#2 in Fig. 8). Instead, in the latter cells doxorubicin was trapped in endolysosomal vesicles and colocalized with Pgp, as visible by colocalization of Pgp-EGFP (green) with LysoTracker (blue) and doxorubicin (red) (Fig. 8, white frames). Thus, lysosomal trapping contributed to protecting the nuclei from the cytotoxic doxorubicin.

### Intercellular transfer of Pgp in RBE4 cells

**Fig. 9:**
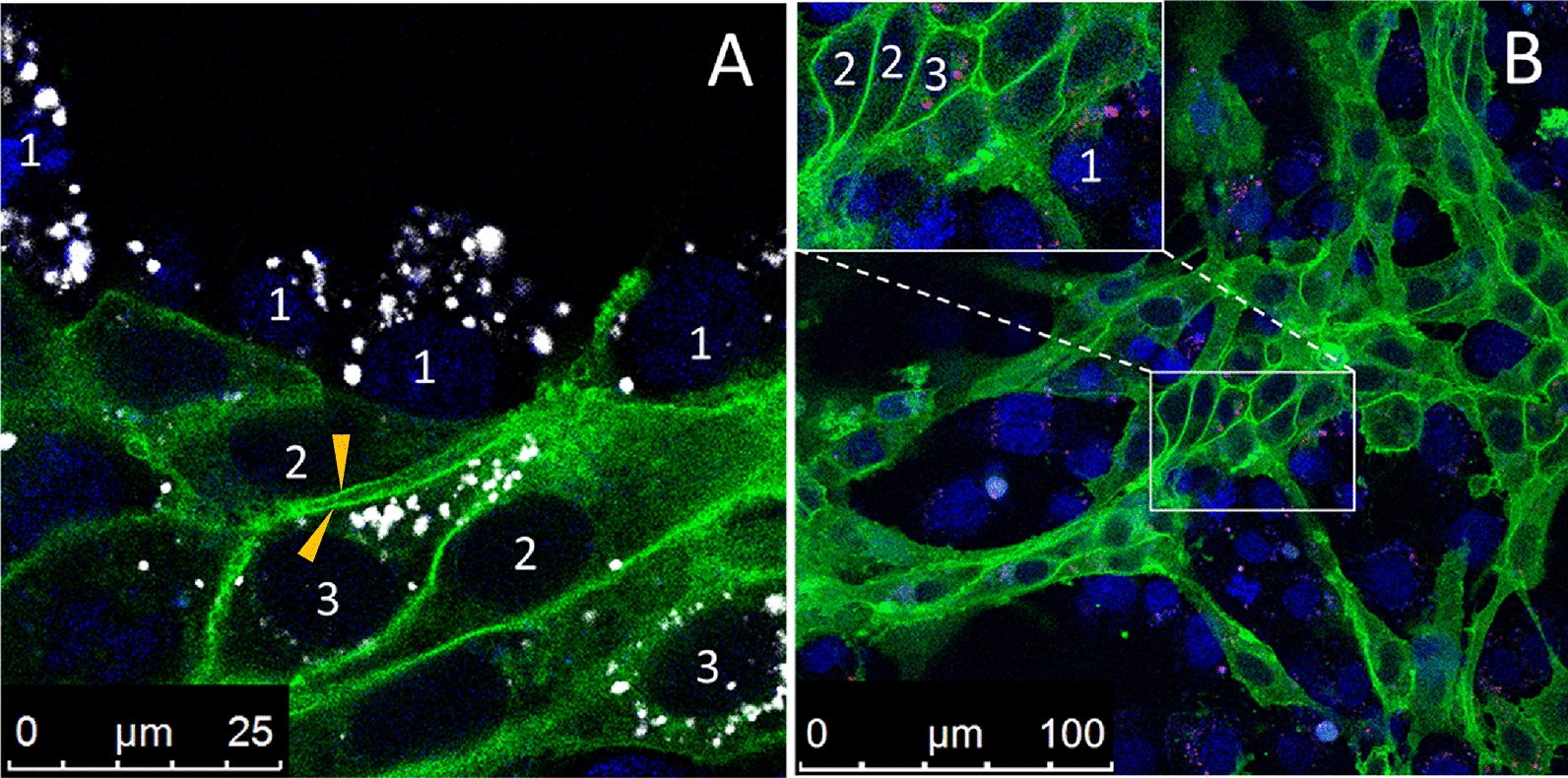
P-glycoprotein transfer from RBE4-*MDR1*-EGFP cells to RBE4 wildtype cells in cocultures. RBE4 wild type cells were labeled with either eFluor 670 (white) or CMTPX (red) and cocultured with an equal amount of RBE4-*MDR1*-EGFP cells until confluency. Cocultures were assessed by confocal laser scanning microscopy and live-cell imaging. Pgp is visible by the EGFP-tag (green) and nuclei are counterstained in blue by bisbenzimide H. The insets show RBE4-WT cells (#1) labeled by the eFluor 670 (**A**) or CMTPX (**B**) staining as well as RBE4-*MDR1*-EGFP cells (#2) visualized by the *MDR1*-EGFP fluorescence and RBE4-WT cells that received Pgp-EGFP from Pgp-EGFP cells (#3). Individual membranes of neighboring cells are representatively labeled (orange arrowheads in **A**). Highlighted is a Pgp containing membrane of an *MDR1* cell close to the membrane of a WT cell after Pgp-EGFP transfer (as indicated by the green fluorescence). Similar experiments with similar outcomes were previously performed with cocultures of wildtype and *MDR1*-EGFP-transduced hCMEC/D3 cells [32], therefore these experiments were not repeated and again illustrated here

In cocultures of hCMEC/D3-WT and hCMEC/D3-*MDR1*-EGFP cells, we have previously shown that the Pgp-EGFP fusion protein was transferred from donor to recipient (WT) cells by cell-to-cell contact and Pgp-EGFP enriched vesicles, which were exocytosed by donor cells and endocytosed by adherent recipient cells [32]. In these previous experiments, such intercellular trafficking was not only studied by confocal laser scanning microscopy (as done here) but also by flow cytometry with the Pgp substrate eFLUXX-ID Gold, by which we demonstrated that the transferred Pgp is functional in the recipient cells [32]. In the present study, we examined whether a cell-to-cell transfer of Pgp-EGFP also occurs in RBE4 cells. For this purpose, RBE4-WT cells were labeled with the vital fluorescence dye eFluor 670 (Fig. 9A) or CMTPX red (Fig. 9B) before coculturing with RBE4-*MDR1*-EGFP cells. The pre-labeling of WT cells before setting up the coculture allows differentiating WT and *MDR1*-EGFP-transduced cells in the monolayer. In the absence of cytotoxic compounds, Pgp was mainly localized at the plasma membrane of RBE4-*MDR1*-EGFP cells (#2 in Fig. 9A, B). The fate of Pgp-EGFP, which is exclusively expressed by *MDR1*-EGFP and not by WT cells, in the coculture could be followed by the green EGFP tag. Thus, Pgp-EGFP fluorescence signal in WT cells (#3 in Fig. 9A, B) visualized by live-cell imaging 2 days after coculturing, indicates intercellular Pgp protein transfer from Pgp-EGFP overexpressing RBE4-*MDR1*-EGFP cells (#2 in Fig. 9A, B) to RBE4-WT cells (#1 in Fig. 9A, B).

### Inhibition of Pgp increases toxicity of doxorubicin in *MDR1*-EGFP transduced RBE4 and hCMEC/D3 cells

**Fig. 10:**
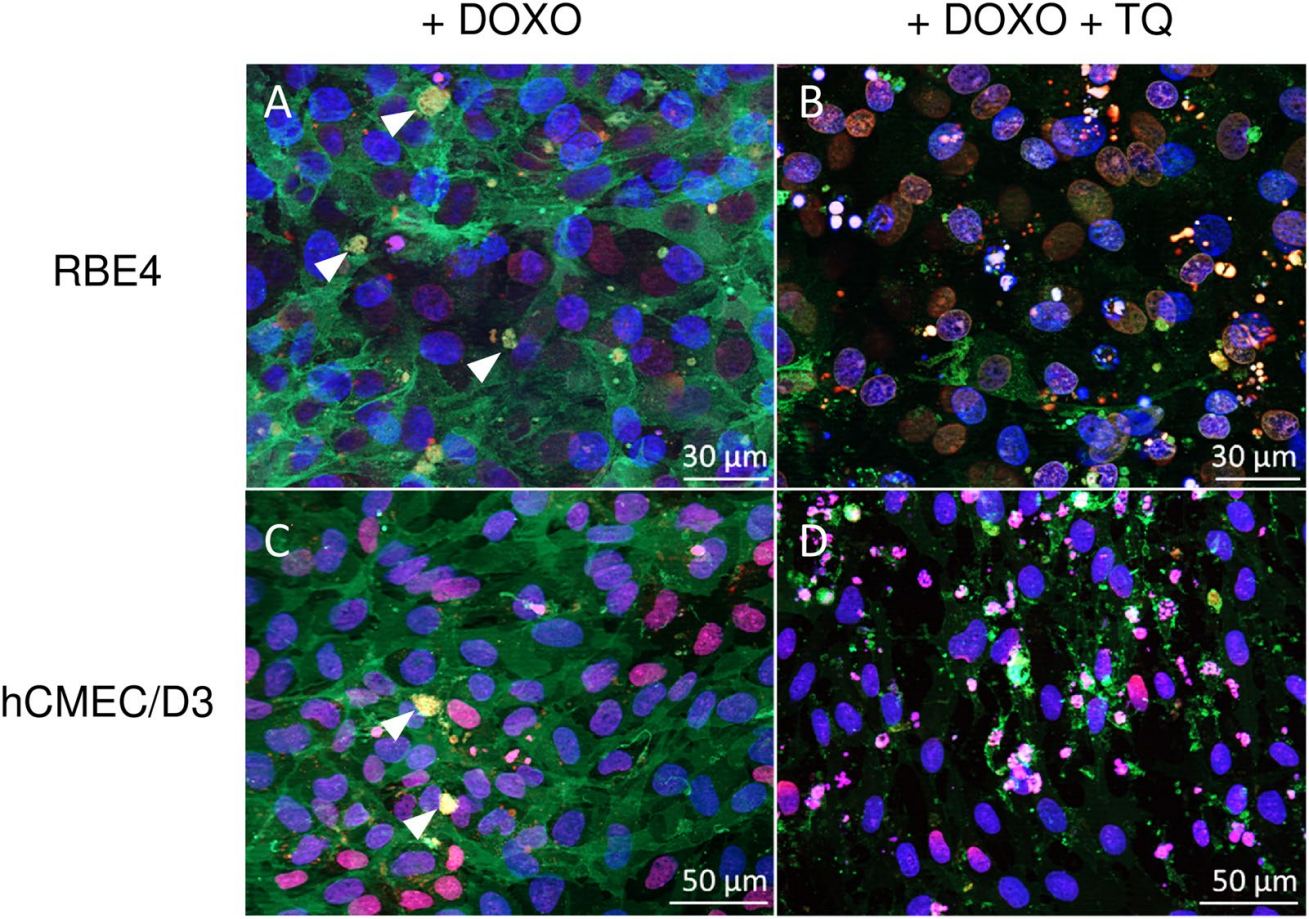
Pgp inhibition with tariquidar (TQ) leads to cell death after doxorubicin (DOXO) treatment. hCMEC/D3 and RBE4 cells expressing Pgp-EGFP (green) show a confluent monolayer 24 h after treatment with 10 µM DOXO (red) (**A**, **C**) as assessed after fixation of the cells and image acquisition using a Zeiss Axio Observer. Inhibition of Pgp by the addition of TQ (0.5 µM) to the cultures leads to cell death (**B**, **D**) as visible by defragmentation of cell nuclei and break down of the monolayer. All images are taken 24 h after DOXO treatment. In the absence of tariquidar (**A**, **C**), yellow aciniform vesicle aggregates (arrowheads in **A** and **C**) were observed in both cell lines that—as indicated by the yellow color—contained both Pgp-EGFP and DOXO. Such drug-sequestering vesicular (lysosomal) aggregates were recently described and characterized by us in more detail and termed “barrier bodies” [13] (see text). Note that the photomicrographs shown in Fig. 8 were taken immediately after 30 min exposure to DOXO, whereas the photomicrographs shown here were taken 24 h after 30 min DOXO exposure, which explains that more cytoplasmic Pgp was seen in the latter experiment shown here

As further proof that Pgp is functional in the *MDR1*-EGFP transduced cells, cells were exposed to the Pgp inhibitor tariquidar (0.5 µM), followed by the addition of doxorubicin (10 µM). As shown in Fig. 10, inhibition of Pgp led to doxorubicin-induced cell death 24 h after exposure (B,D) as visible by defragmentation of cell nuclei and break down of the monolayer, whereas in the absence of Pgp inhibition, cells showed a confluent monolayer without any signs of toxicity upon exposure to doxorubicin (A,C). Interestingly, in the absence of tariquidar, yellow aciniform vesicle aggregates were observed in both cell lines that—as indicated by the yellow color—contained both Pgp-EGFP (green) and doxorubicin (red). Such drug-sequestering vesicular (lysosomal) aggregates were recently described and characterized by us in more detail and termed “barrier bodies”, as they are attached to the apical side of the plasma membrane and phagocytized by neutrophils, thus constituting a new mechanism of drug disposal at the BBB [13].

### Lysosomotropic effect of doxorubicin in *MDR1*-EGFP transduced RBE4 and hCMEC/D3 cells

**Fig. 11:**
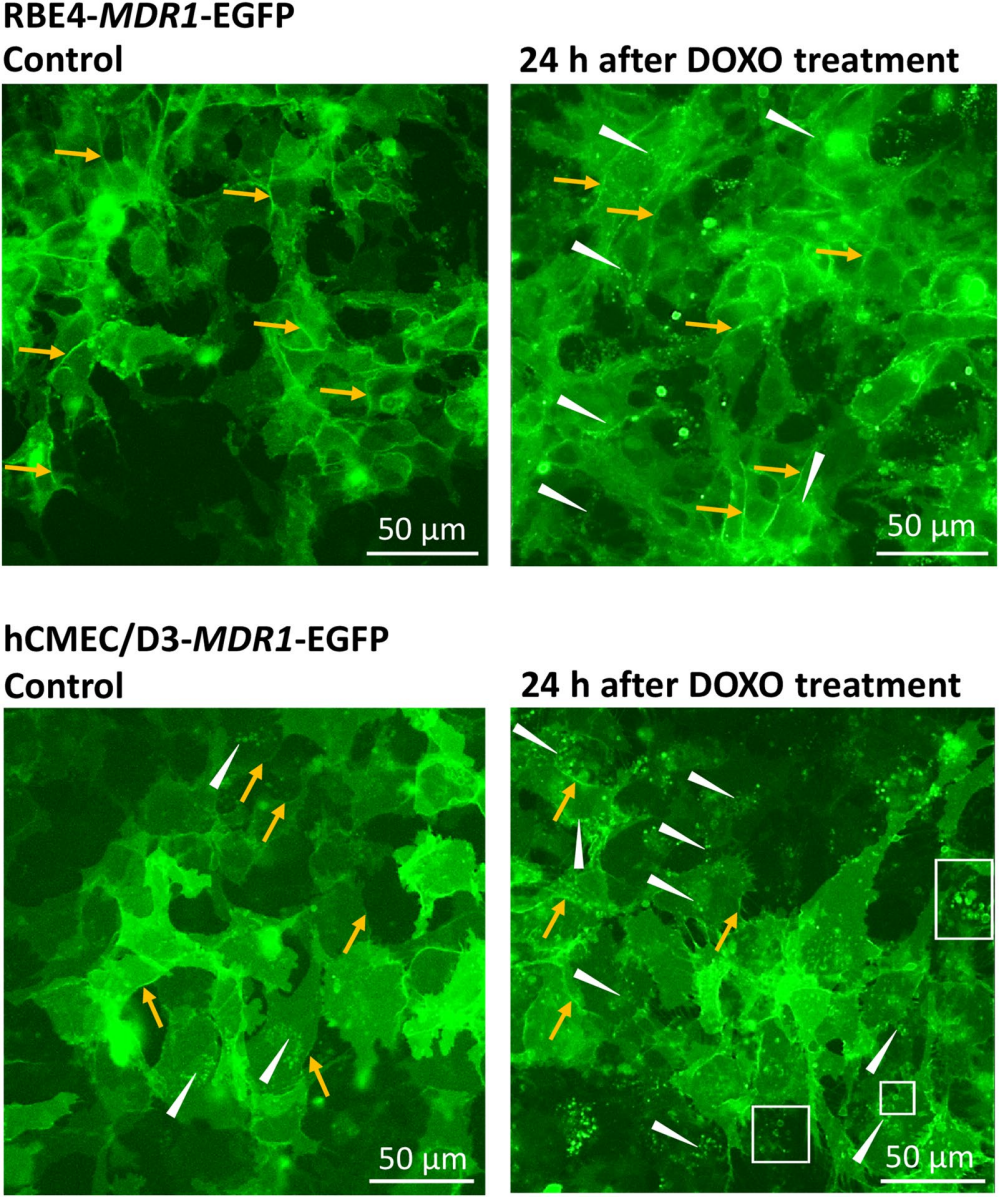
Increased number and size of intracellular Pgp-EGFP-positive vesicles after treatment of RBE4-*MDR1*-EGFP or hCMEC/D3-*MDR1*-EGFP cells with doxorubicin (DOXO). Comparison of the number and size of intracellular Pgp-EGFP positive vesicles in RBE4- and hCMEC/D3-*MDR1*-EGFP cells before (control) and 24 h after treatment with DOXO (10 µM). Live-cell image acquisition was performed with an inverted fluorescence Lumascope 620 in the cell culture incubator. In both cell lines, Pgp-EGFP (green fluorescent) was localized in the cell membrane (orange arrows), whereas Pgp-positive intracellular vesicles (white arrows), most likely of endolysosomal origin, were seen in hCMEC/D3 but hardly in RBE4 cells (similar to the confocal laser scanning photomicrographs shown in Fig. 1). Exposure to DOXO increased the number of Pgp-EGFP positive vesicles in both cell lines. Furthermore, the size of the Pgp-EGFP positive vesicles appeared larger after DOXO exposure (white boxes). An increase of the number of intracellular green-fluorescent vesicles after DOXO treatment may indicate biogenesis of Pgp-containing intracellular vesicles and/or transport of Pgp into the vesicles. Those mechanisms likely enhance drug sequestration capacity of the cells after exposure to cytotoxic compounds/Pgp-substrates. See Additional file 5 for quantification of Pgp-EGFP positive vesicles before and after treatment with DOXO

Doxorubicin is known to exert lysosomotropic effects [12], which prompted us to examine such effects in *MDR1*-EGFP transduced RBE4 and hCMEC/D3 cells. For this purpose, we performed live-cell image acquisition with a fluorescence microscope (Lumascope 620) within the cell culture incubator before and after exposure to doxorubicin. These results indicated that doxorubicin increased the number of Pgp-EGFP-positive intercellular vesicles in both cell lines (Fig. 11). This was substantiated by counting these vesicles of likely endolysosomal origin (Additional file 5). Exposure to doxorubicin (10 µM) increased the average number of Pgp-EGFP-positive vesicles per cell by 220% in RBE4 cells and 170% in hCMEC/D3 cells, respectively. In addition to the number, the size of the Pgp-EGFP-positive vesicles seemed to increase (Fig. 11). The increase in number and size of EGFP-positive intracellular vesicles after treatment with doxorubicin may either indicate lysosomal biogenesis or fusion as reported previously for exposure of other cell types to chemotherapeutic drugs such as doxorubicin [12]. Alternatively, the increased number of EGFP-positive intracellular vesicles may indicate increased transport of Pgp-EGFP into the vesicles, which, however, is considered less likely. As noted above, we have previously shown for hCMEC/D3 cells that the EGFP-positive intracellular vesicles are endolysosomal vesicles [13, 31], which was confirmed in the present experiments for RBE4 cells (Fig. 8).

## Discussion

We have shown previously that stable transduction of hCMEC/D3 cells with a doxycycline-inducible *MDR1*-EGFP fusion plasmid allows studying the intracellular localization and intra- as well as intercellular trafficking of Pgp-EGFP in an in vitro model of the human BBB [13, 31, 32]. Pgp expression can be effectively controlled in this system, providing an ideal environment in which cellular trafficking of the protein and interactions with a variety of intracellular targets can be studied [9]. Furthermore, as shown in our previous studies, hCMEC/D3-*MDR1*-EGFP cells can be used as a tool to study the effects of drugs on Pgp expression and functionality in human BCECs [9]. Since the Pgp is artificially overexpressed in hCMEC/D3-*MDR1*-EGFP cells, it was important to demonstrate that intrinsic and drug-induced Pgp trafficking was not simply a consequence of gross overexpression of the transporter, which was done recently by showing the same processes in primary cultured porcine BCECs [13].

In the present study, we used the *MDR1*-EGFP transduction strategy for RBE4 cells, i.e., one of the best characterized and commonly used immortalized rat BCEC lines in BBB modeling [17, 24, 25, 29, 53, 54]. The major aim of the present study was to perform a face-to-face comparison of *MDR1*-EGFP-transduced RBE4 vs. hCMEC/D3 cells to evaluate how the BCEC type (rat vs. human) and the resulting species differences in the cellular environment of Pgp affect the localization, trafficking, and function of Pgp from the same species (human). Interestingly, the Pgp-EGFP fusion protein was expressed differently in both BCEC types in that substantially more intracellular localization of Pgp-EGFP was seen in hCMEC/D3 cells, whereas the fusion protein was almost exclusively expressed at the plasma membrane in RBE4 cells. However, exposure to doxorubicin led to endolysosomal localization of both Pgp-EGFP and doxorubicin in RBE4-*MDR1*-EGFP cells, indicating that doxorubicin exposure induced the formation of Pgp-containing endolysosomal vesicles in RBE4 cells as previously reported for cancer cells [12]. Furthermore, these data suggest that Pgp-mediated lysosomal sequestration of this chemotherapeutic drug occurs in BCECs as recently reported for hCMEC/D3-*MDR1*-EGFP cells [13].

Except for red blood cells, all eukaryotic cells, including BBB endothelial cells, contain lysosomes, but their structure and number vary depending on the cell type and functional state [55, 56]. Lysosomes are highly acidic, membrane-enclosed, intracellular organelles, carrying a battery of hydrolytic enzymes as well as several membrane-associated proteins [55]. Recent evidence indicates that the importance of the lysosome for cellular homeostasis goes far beyond the simple degradation of cell waste [43, 57, 58]. Lysosomes can rapidly adapt to both intracellular and extracellular cues and control their biogenesis [57]. Two main mediators of these lysosomal adaptation mechanisms are the mechanistic target of rapamycin (mTOR) kinase complex and the transcription factor EB (TFEB) [12, 57], which will be discussed in more detail below. As described in the Introduction, by sequestration of anticancer drugs, lysosomes play a role in the chemoresistance of cancer cells [10–12]. We have recently described similar mechanisms of lysosomal drug sequestration in immortalized and primary cultured BCECs [13], indicating that lysosomes may contribute to the function of the BBB in protecting the brain from intoxication by xenobiotics [9]. In this respect, it is important to note that lysosomal drug sequestration in cells of the BBB is not restricted to chemotherapeutics, but also occurs with other drugs, including the Pgp substrate, dLop [42], which was used here in some of the vectorial transport experiments.

As indicated by the present data, lysosomal trapping of doxorubicin in RBE4-*MDR1*-EGFP cells likely contributed to the protection of the cell nucleus from damage by this cytotoxic agent, whereas doxorubicin bound to its nuclear target in RBE4-WT cells, inducing cell death. In the lysosomes, doxorubicin was colocalized with Pgp-EGFP, indicating that Pgp, at least in part, mediated the lysosomal sequestration of doxorubicin as recently reported for hCMEC/D3-*MDR1*-EGFP cells [13] and cancer cells [59]. As described in the Introduction, cytostatic agents enter the lysosome either by passive diffusion along the pH gradient or may be actively transported across the membrane by inward-turned Pgp embedded in the lysosomal membrane [11, 12], although the latter mechanism is debated [51, 60]. The fate of drugs sequestered in lysosomes remains to be elucidated in more detail, but it was suggested that they either stay trapped in lysosomes, are degraded in the lysosomes, or are eliminated from the cell by drug-induced lysosomal exocytosis, preventing lysosomal damage [9, 12]. Furthermore, we have recently described a new mechanism of drug disposal by hCMEC/D3 and primary porcine BCECs, which is the shedding of lysosomal Pgp/substrate complexes (“barrier bodies”) at the apical membrane and subsequent phagocytosis by neutrophils [13]. Such aciniform barrier bodies were also observed in the present experiments in *MDR1*-EGFP transduced RBE4 cells. The contribution of lysosomes to chemoresistance has raised interest in lysosome-targeting strategies to sensitize tumor cells to chemotherapy [12]. Based on our findings on lysosomal trapping in BCECs that form the BBB, we have proposed lysosome-targeting strategies to enhance drug delivery to the brain [9]. As suggested by the present data, *MDR1*-EGFP transduced RBE4 cells provide an interesting tool to study such strategies.

Lysosomal drug accumulation is known to trigger cytoplasmic to nuclear translocation of the transcription factor TFEB, i.e., the master transcriptional regulator of lysosomal biogenesis and autophagy [11, 12, 57]. TFEB activation results in increased lysosomal biogenesis, an elevated number of lysosomes per cell, increased lysosomal drug sequestration, and consequent drug resistance. By accumulating in the lysosomal lumen, cytostatic weak bases such as doxorubicin act like classic lysosomotropic compounds, raising lysosomal pH and increasing lysosomal volume, which activates TFEB. Furthermore, such drugs may directly activate TFEB, thus leading to the formation of lysosomes [12] as shown here. Furthermore, TFEB acts as a transcription factor for several proteins that are essential for autophagy, which is a complex process promoting cell survival during stress conditions and a driving factor for the chemoresistance of cancer cells [12]. We have recently suggested that these processes also play a role in the adaptation of the BBB to high blood levels of potentially toxic xenobiotics, thereby protecting the brain from intoxication [9]. *MDR1*-EGFP transduced RBE4 cells appear to provide a useful model to study these processes.

The use of fluorescent substrates, such as Rho123, is valuable for the evaluation of membrane transporter activity [14, 17, 61]. In the present study, the functionality of Pgp in WT and *MDR1*-EGFP transduced RBE4 and hCMEC/D3 cells as well as in primary rBCECs could be demonstrated by the Rho123 accumulation assay. This assay showed that transduction with *MDR1*-EGFP significantly increased the efflux of Rho123, reaching values that were also determined in primary rBCECs. Using the Rho123 uptake assay, we have previously compared RBE4- and hCMEC/D3-WT cells in their response to various known Pgp inducers, such as dexamethasone and several cytostatic drugs (including doxorubicin), as well as antiseizure drugs [36]. Known Pgp inducers increased Rho123 efflux in both cell lines, but marked inter-cell line differences in effect size were observed. RBE4 cells were much more sensitive to the Pgp inducing effects of dexamethasone, doxorubicin, and puromycin than hCMEC/D3 cells. In rBCECs, Rho123 accumulation was not affected by exposure with dexamethasone but was significantly reduced by puromycin [36]. At least in part, the differences between RBE4 and hCMEC/D3 cells could be due to species differences in the expression and functionality of nuclear receptors that mediate drug effects on Pgp in BCECs [62]. For instance, stimulation of the vitamin D receptor, a hormone nuclear receptor, increased Pgp expression 2.5-fold in RBE4 cells, threefold in hCMEC/D3 cells, but fourfold in isolated rat brain capillaries [63].

Veszelka et al. [21] compared the expression of selected BBB related genes including tight junction proteins, solute carriers (SLC), ABC transporters, and metabolic enzymes in several epithelial cell and BCEC lines, including RBE4 and hCMEC/D3 cells, as well as, for reference, primary rBCECs that were cocultured with astrocytes and pericytes (E: endothelial cells P: pericytes, A: astrocytes, EPA). Furthermore, they immunostained tight junction proteins and studied transporter functionality by drug transport experiments in the Transwell system. The mRNA expression level of occludin was high in the EPA and hCMEC/D3 cells but low in the rat RBE4 cell line. Furthermore, marked differences in mRNA expression of claudins were found between RBE4, hCMEC/D3, and EPA cells. The mRNA level of claudin-5, which significantly contributes to the tightness of BCECs [64], was significantly higher in the primary EPA model than in any of the cell lines [21]. Furthermore, claudin-5 protein immunostaining was well visible on the cell border of primary rBCECs, while it was very weak or undetectable in the immortalized BCEC lines. All three BBB models (EPA, RBE4, hCMEC/D3) expressed the mRNA of Pgp with the highest values in EPA and lowest values in RBE4 [21]. Average TEER values of RBE4 (64 Ω cm^2^) and hCMEC/D3 cells (45 Ω cm^2^) obtained with chopstick electrode measurement were markedly lower than the TEER value of EPA (475 Ω cm^2^) and this was associated with high paracellular flux of fluorescein in the immortalized BCEC lines [21]. Therefore, RBE4 and hCMEC/D3 cells were excluded from drug transport studies because they did not form a restrictive paracellular barrier to allow screening of the permeability of small molecules.

The low TEER and high paracellular permeability of RBE4 and hCMEC/D3 were confirmed in the present study and, as expected, this was not altered by transduction with *MDR1*-EGFP. As a consequence, no vectorial drug transport of Pgp substrates was observed in these cell lines, whereas such transport was determined in primary rBCECs and *MDR1*-transfected LLC cells, which are widely used for Pgp screening of NCEs as a surrogate in vitro model of the BBB [14, 17–20]. We have shown recently that transduction of hCMEC/D3 cells with claudin-5 increases TEER and reduces paracellular mannitol flux in these cells; however, values of primary cultured BCECs were not reached [43].

RBE4 cells have previously been used for transfection with various plasmids, including a protein tyrosine kinase [65], green fluorescent magnetic nanoparticles [66], mCherry-*Mct1* (monocarboxylic acid transporter 1 [67]), siRNA-chitosan to silence Pgp [68], choline acetyltransferase [69], interleukin 15 receptor splicing variants [70], and mutated versions of *MDR1* [71]. In the latter study, transfection of RBE4 cells with mutated versions of *MDR1*, in the caveolin-1 interaction motif, decreased the interaction between Pgp and caveolin-1 and enhanced Pgp transport activity and cell migration.

One of the inherent shortcomings of in vitro BBB models that use cell-based assays in a two-compartment Transwell chamber is the lack of critical microenvironmental parameters such as hemodynamic shear stress [26]. Prabhakarpandian et al. [72] described a two-compartment chamber microfluidic-based synthetic microvasculature model of the BBB (SyMBBB), in which RBE4 cells were cultured in the apical compartment under fluidic shear conditions and in continuous contact with astrocyte-conditioned medium in the basolateral compartment. In this model, compared to RBE4 cells in Transwell chambers, tight junction proteins (claudin-1 and ZO-1) and Pgp were significantly upregulated, resulting in a decreased paracellular flux of fluorescein isothiocyanate-dextran and increased efflux of Rho123 [72]. However, it was not studied whether the SyMBBB model allows determining vectorial drug transport. It would be interesting to use this system with the *MDR1*-EGFP transduced RBE4 cells presented here. Several other studies have reported that coculturing of RBE4 cells with astrocytes or use of astrocyte-conditioned medium increases barrier properties of the RBE4 cells [27, 29, 73–78], but none of these studies demonstrated that this allow studying vectorial drug transport. Our experiments with coculturing of hCMEC/D3 cells with astrocytes failed to demonstrate vectorial transport of Pgp substrates [79].

## Conclusions

Overall, the present data indicate that *MDR1*-EGFP transduced RBE4 cells are an interesting tool to study lysosome biogenesis and Pgp-mediated lysosomal drug trapping in response to chemotherapeutic agents and other compounds. Furthermore, RBE4 cells have proved useful, in parallel with primary cultures of rBCECs, for various cellular approaches towards the understanding of BBB biology [17, 24, 25, 53, 54, 80]. In addition, like MDR1-EGFP transduced hCMEC/D3 cells [13, 31, 32], *MDR1*-EGFP transduced RBE4 cells provide an ideal tool to study Pgp trafficking at the level of the BBB. However, similar to hCMEC/D3 cells, using a conventional Transwell assay, WT or *MDR1*-EGFP transduced RBE4 cells do not generate the necessary restrictive paracellular barrier properties that would allow using them in transendothelial permeability screening of small drugs. This problem may be resolved by using dynamic, hollow fiber-based models or microfluidic models, which incorporate the influence of shear stress, thus strengthening the barrier properties of the BCEC monolayers [19].

## Supplementary Information

**Additional file 1.** Influence of insert characteristics (Transwell® vs. ThinCert® and TC‐insert system) on monolayer formation and barrier integrity was assessed by comparison of transendothelial electrical resistance (TEER) and mannitol permeability of hCMEC/D3 and RBE4 cells.

**Additional file 2.** Cell morphology of RBE4 and hCMEC/D3 wildtype and *MDR1*-EGFP expressing cells.

**Additional file 3.** Morphology of primary rat brain capillary endothelial cells (rBCECs) and immortalized LLC-*MDR1* kidney epithelial cells.

**Additional file 4.** P-glycoprotein localization in *MDR1*-EGFP expressing RBE4 and hCMEC/D3 cells.

**Additional file 5.** Pgp-EGFP-positive intracellular vesicles in RBE4 and hCMEC/D3 cells before and after exposure to doxorubicin (DOXO).

**Additional file 6.** Indirect staining of Pgp in *MDR1*-EGFP transduced RBE4 and hCMEC/D3 cells colocalizes with the Pgp-EGFP fluorescence signal.

**Additional file 7.** Pgp localization in RBE4-WT cells by indirect Pgp staining.

## Data Availability

The datasets used and/or analyzed during the current study are available from the corresponding author on reasonable request.

## Abbreviations

BBB: Blood–brain barrier
BCEC: Brain capillary endothelial cell
CETA: Concentration equilibrium transport assay
dLop: *N*-Desmethyl loperamide
EGFP: Enhanced green fluorescent protein
MAF: Multidrug resistance activity factor
MDR1: Multidrug resistance 1
Pgp: P-glycoprotein
Rho123: Rhodamine 123
TEER: Transepithelial/-endothelial electrical resistance
TFEB: Transcription factor EB

## References

[1] Borst P, Schinkel AH (2013). P-glycoprotein ABCB1: a major player in drug handling by mammals. J Clin Invest.

[2] Daneman R, Prat A (2015). The blood-brain barrier. Cold Spring Harb Perspect Biol.

[3] Schinkel AH (1999). P-Glycoprotein, a gatekeeper in the blood-brain barrier. Adv Drug Deliv Rev.

[4] Löscher W, Potschka H (2005). Drug resistance in brain diseases and the role of drug efflux transporters. Nature Rev Neurosci.

[5] Abbott NJ (2013). Blood-brain barrier structure and function and the challenges for CNS drug delivery. J Inherit Metab Dis.

[6] Polli JW, Wring SA, Humphreys JE, Huang L, Morgan JB, Webster LO (2001). Rational use of in vitro P-glycoprotein assays in drug discovery. J Pharmacol Exp Ther.

[7] Feng B, Mills JB, Davidson RE, Mireles RJ, Janiszewski JS, Troutman MD (2008). In vitro P-glycoprotein assays to predict the in vivo interactions of P-glycoprotein with drugs in the central nervous system. Drug Metab Dispos.

[8] Feng B, Doran AC, Di L, West MA, Osgood SM, Mancuso JY (2018). Prediction of human brain penetration of P-glycoprotein and breast cancer resistance protein substrates using in vitro transporter studies and animal models. J Pharm Sci.

[9] Löscher W, Gericke B (2020). Novel intrinsic mechanisms of active drug extrusion at the blood-brain barrier: potential targets for enhancing drug delivery to the brain?. Pharmaceutics.

[10] Seebacher N, Lane DJ, Richardson DR, Jansson PJ (2016). Turning the gun on cancer: utilizing lysosomal P-glycoprotein as a new strategy to overcome multi-drug resistance. Free Radic Biol Med.

[11] Zhitomirsky B, Assaraf YG (2016). Lysosomes as mediators of drug resistance in cancer. Drug Resist Updat.

[12] Geisslinger F, Müller M, Vollmar AM, Bartel K (2020). Targeting lysosomes in cancer as promising strategy to overcome chemoresistance-a mini review. Front Oncol.

[13] Noack A, Gericke B, von Köckritz-Blickwede M, Menze A, Noack S, Gerhauser I (2018). A novel mechanism of drug extrusion by brain endothelial cells via lysosomal drug trapping and disposal by neutrophils. Proc Natl Acad Sci (USA).

[14] Schwab D, Fischer H, Tabatabaei A, Poli S, Huwyler J (2003). Comparison of in vitro P-glycoprotein screening assays: recommendations for their use in drug discovery. J Med Chem.

[15] Gumbleton M, Audus KL (2001). Progress and limitations in the use of in vitro cell cultures to serve as a permeability screen for the blood-brain barrier. J Pharm Sci.

[16] Wilhelm I, Krizbai IA (2014). In vitro models of the blood-brain barrier for the study of drug delivery to the brain. Mol Pharm.

[17] Gameiro M, Silva R, Rocha-Pereira C, Carmo H, Carvalho F, Bastos ML (2017). Cellular models and in vitro assays for the screening of modulators of P-gp, MRP1 and BCRP. Molecules.

[18] Fischer H, Senn C, Ullah M, Cantrill C, Schuler F, Yu L (2021). Calculation of an apical efflux ratio from P-glycoprotein (P-gp) in vitro transport experiments shows an improved correlation with in vivo cerebrospinal fluid measurements in rats: impact on P-gp screening and compound optimization. J Pharmacol Exp Ther.

[19] Neuhaus W (2021). In vitro models of the blood-brain barrier. Handb Exp Pharmacol.

[20] Patel NC, Feng B, Hou X, West MA, Trapa PE, Sciabola S (2021). Harnessing preclinical data as a predictive tool for human brain tissue targeting. ACS Chem Neurosci.

[21] Veszelka S, Tóth A, Walter FR, Tóth AE, Gróf I, Mészáros M (2018). Comparison of a rat primary cell-based blood-brain barrier model with epithelial and brain endothelial cell lines: gene expression and drug transport. Front Mol Neurosci.

[22] Hellinger E, Veszelka S, Toth AE, Walter F, Kittel A, Bakk ML (2012). Comparison of brain capillary endothelial cell-based and epithelial (MDCK-MDR1, Caco-2, and VB-Caco-2) cell-based surrogate blood-brain barrier penetration models. Eur J Pharm Biopharm.

[23] Helms HC, Abbott NJ, Burek M, Cecchelli R, Couraud PO, Deli MA (2016). In vitro models of the blood-brain barrier: an overview of commonly used brain endothelial cell culture models and guidelines for their use. J Cereb Blood Flow Metab.

[24] Rahman NA, Rasil AN, Meyding-Lamade U, Craemer EM, Diah S, Tuah AA (2016). Immortalized endothelial cell lines for in vitro blood-brain barrier models: a systematic review. Brain Res.

[25] Kaisar MA, Sajja RK, Prasad S, Abhyankar VV, Liles T, Cucullo L (2017). New experimental models of the blood-brain barrier for CNS drug discovery. Expert Opin Drug Discov.

[26] Modarres HP, Janmaleki M, Novin M, Saliba J, El Hajj F, RezayatiCharan M (2018). In vitro models and systems for evaluating the dynamics of drug delivery to the healthy and diseased brain. J Control Release.

[27] Roux F, Durieu-Trautmann O, Chaverot N, Claire M, Mailly P, Bourre JM (1994). Regulation of gamma-glutamyl transpeptidase and alkaline phosphatase activities in immortalized rat brain microvessel endothelial cells. J Cell Physiol.

[28] Weksler BB, Subileau EA, Perrière N, Charneau P, Holloway K, Leveque M (2005). Blood-brain barrier-specific properties of a human adult brain endothelial cell line. FASEB J.

[29] Roux F, Couraud PO (2005). Rat brain endothelial cell lines for the study of blood-brain barrier permeability and transport functions. Cell Mol Neurobiol.

[30] Urich E, Lazic SE, Molnos J, Wells I, Freskgard PO (2012). Transcriptional profiling of human brain endothelial cells reveals key properties crucial for predictive in vitro blood-brain barrier models. PLoS ONE.

[31] Noack A, Noack S, Hoffmann A, Maalouf K, Buettner M, Couraud PO (2014). Drug-induced trafficking of p-glycoprotein in human brain capillary endothelial cells as demonstrated by exposure to mitomycin C. PLoS ONE.

[32] Noack A, Noack S, Buettner M, Naim HY, Löscher W (2016). Intercellular transfer of P-glycoprotein in human blood-brain barrier endothelial cells is increased by histone deacetylase inhibitors. Sci Rep.

[33] Sharom FJ (2014). Complex interplay between the P-glycoprotein multidrug efflux pump and the membrane: its role in modulating protein function. Front Oncol.

[34] Chu X, Bleasby K, Evers R (2013). Species differences in drug transporters and implications for translating preclinical findings to humans. Expert Opin Drug Metab Toxicol.

[35] Harayama T, Riezman H (2018). Understanding the diversity of membrane lipid composition. Nat Rev Mol Cell Biol.

[36] Alms D, Fedrowitz M, Römermann K, Noack A, Löscher W (2014). Marked differences in the effect of antiepileptic and cytostatic drugs on the functionality of P-glycoprotein in human and rat brain capillary endothelial cell lines. Pharm Res.

[37] Régina A, Romero IA, Greenwood J, Adamson P, Bourre JM, Couraud PO (1999). Dexamethasone regulation of P-glycoprotein activity in an immortalized rat brain endothelial cell line. GPNT J Neurochem.

[38] Perrière N, Demeuse P, Garcia E, Regina A, Debray M, Andreux JP (2005). Puromycin-based purification of rat brain capillary endothelial cell cultures. Effect on the expression of blood-brain barrier-specific properties. J Neurochem.

[39] Artursson P (1990). Epithelial transport of drugs in cell culture. I: A model for studying the passive diffusion of drugs over intestinal absorptive (Caco-2) cells. J Pharm Sci.

[40] Huber O, Brunner A, Maier P, Kaufmann R, Couraud PO, Cremer C (2012). Localization microscopy (SPDM) reveals clustered formations of P-glycoprotein in a human blood-brain barrier model. PLoS ONE.

[41] Luna-Tortós C, Fedrowitz M, Löscher W (2008). Several major antiepileptic drugs are substrates for human P-glycoprotein. Neuropharmacology.

[42] Kannan P, Brimacombe KR, Kreisl WC, Liow JS, Zoghbi SS, Telu S (2011). Lysosomal trapping of a radiolabeled substrate of P-glycoprotein as a mechanism for signal amplification in PET. Proc Natl Acad Sci U S A.

[43] Gericke B, Römermann K, Noack A, Noack S, Kronenberg J, Blasig IE (2020). A face-to-face comparison of claudin-5 transduced human brain endothelial (hCMEC/D3) cells with porcine brain endothelial cells as blood-brain barrier models for drug transport studies. Fluids Barriers CNS.

[44] McInerney MP, Pan Y, Short JL, Nicolazzo JA (2017). Development and validation of an in-cell western for quantifying p-glycoprotein expression in human brain microvascular endothelial (hCMEC/D3) cells. J Pharm Sci.

[45] Eustaquio DI, Lye P, Bloise E, Matthews SG (2021). Function of Multidrug Resistance Transporters is Disrupted by Infection Mimics in Human Brain Endothelial Cells. Tissue Barriers.

[46] Régina A, Koman A, Piciotti M, El Hafny B, Center MS, Bergmann R (1998). Mrp1 multidrug resistance-associated protein and P-glycoprotein expression in rat brain microvessel endothelial cells. J Neurochem.

[47] Eigenmann DE, Xue G, Kim KS, Moses AV, Hamburger M, Oufir M (2013). Comparative study of four immortalized human brain capillary endothelial cell lines, hCMEC/D3, hBMEC, TY10, and BB19, and optimization of culture conditions, for an in vitro blood-brain barrier model for drug permeability studies. Fluids Barriers CNS.

[48] Mi Y, Mao Y, Cheng H, Ke G, Liu M, Fang C (2020). Studies of blood-brain barrier permeability of gastrodigenin in vitro and in vivo. Fitoterapia.

[49] Wuest DM, Wing AM, Lee KH (2013). Membrane configuration optimization for a murine in vitro blood-brain barrier model. J Neurosci Methods.

[50] Kannan P, Brimacombe KR, Zoghbi SS, Liow JS, Morse C, Taku AK (2010). N-desmethyl-loperamide is selective for P-glycoprotein among three ATP-binding cassette transporters at the blood-brain barrier. Drug Metab Dispos.

[51] Stefan SM, Jansson PJ, Kalinowski DS, Anjum R, Dharmasivam M, Richardson DR (2020). The growing evidence for targeting P-glycoprotein in lysosomes to overcome resistance. Future Med Chem.

[52] Mahoney BP, Raghunand N, Baggett B, Gillies RJ (2003). Tumor acidity, ion trapping and chemotherapeutics I Acid pH affects the distribution of chemotherapeutic agents in vitro. Biochem Pharmacol.

[53] Bendayan R, Lee G, Bendayan M (2002). Functional expression and localization of P-glycoprotein at the blood brain barrier. Microsc Res Tech.

[54] Aschner M, Fitsanakis VA, dos Santos AP, Olivi L, Bressler JP (2006). Blood-brain barrier and cell-cell interactions: methods for establishing in vitro models of the blood-brain barrier and transport measurements. Methods Mol Biol.

[55] Xu H, Ren D (2015). Lysosomal physiology. Annu Rev Physiol.

[56] Toth AE, Holst MR, Nielsen MS (2020). Vesicular transport machinery in brain endothelial cells: what we know and what we do not. Curr Pharm Des.

[57] Settembre C, Ballabio A (2014). Lysosomal adaptation: how the lysosome responds to external cues. Cold Spring Harb Perspect Biol.

[58] Trivedi PC, Bartlett JJ, Pulinilkunnil T (2020). Lysosomal biology and function: modern view of cellular debris bin. Cells.

[59] Yamagishi T, Sahni S, Sharp DM, Arvind A, Jansson PJ, Richardson DR (2013). P-glycoprotein mediates drug resistance via a novel mechanism involving lysosomal sequestration. J Biol Chem.

[60] Szakacs G, Abele R (2020). An inventory of lysosomal ABC transporters. FEBS Lett.

[61] Fontaine M, Elmquist WF, Miller DW (1996). Use of rhodamine 123 to examine the functional activity of P-glycoprotein in primary cultured brain microvessel endothelial cell monolayers. Life Sci.

[62] Chan GN, Hoque MT, Bendayan R (2013). Role of nuclear receptors in the regulation of drug transporters in the brain. Trends Pharmacol Sci.

[63] Durk MR, Chan GN, Campos CR, Peart JC, Chow EC, Lee E (2012). 1Î±,25-Dihydroxyvitamin D3-liganded vitamin D receptor increases expression and transport activity of P-glycoprotein in isolated rat brain capillaries and human and rat brain microvessel endothelial cells. J Neurochem.

[64] Greene C, Hanley N, Campbell M (2019). Claudin-5: gatekeeper of neurological function. Fluids Barriers CNS.

[65] Barakat S, Demeule M, Pilorget A, Régina A, Gingras D, Baggetto LG (2007). Modulation of p-glycoprotein function by caveolin-1 phosphorylation. J Neurochem.

[66] Linemann T, Thomsen LB, Jardin KG, Laursen JC, Jensen JB, Lichota J (2013). Development of a novel lipophilic, magnetic nanoparticle for in vivo drug delivery. Pharmaceutics.

[67] Liu Z, Sneve M, Haroldson TA, Smith JP, Drewes LR (2016). Regulation of monocarboxylic acid transporter 1 trafficking by the canonical Wnt/β-catenin pathway in rat brain endothelial cells requires cross-talk with notch signaling. J Biol Chem.

[68] Malmo J, Sandvig A, Varum KM, Strand SP (2013). Nanoparticle mediated P-glycoprotein silencing for improved drug delivery across the blood-brain barrier: a siRNA-chitosan approach. PLoS ONE.

[69] Malo M, Diebler MF, Prado DC, Meunier FM, Dunant Y, Bloc A (1999). Evoked acetylcholine release by immortalized brain endothelial cells genetically modified to express choline acetyltransferase and/or the vesicular acetylcholine transporter. J Neurochem.

[70] Wu X, Pan W, Stone KP, Zhang Y, Hsuchou H, Kastin AJ (2010). Expression and signaling of novel IL15Ralpha splicing variants in cerebral endothelial cells of the blood-brain barrier. J Neurochem.

[71] Barakat S, Turcotte S, Demeule M, Lachambre MP, Régina A, Baggetto LG (2008). Regulation of brain endothelial cells migration and angiogenesis by P-glycoprotein/caveolin-1 interaction. Biochem Biophys Res Commun.

[72] Prabhakarpandian B, Shen MC, Nichols JB, Mills IR, Sidoryk-Wegrzynowicz M, Aschner M (2013). SyM-BBB: a microfluidic Blood Brain Barrier model. Lab Chip.

[73] El Hafny B, Chappey O, Piciotti M, Debray M, Boval B, Roux F (1997). Modulation of P-glycoprotein activity by glial factors and retinoic acid in an immortalized rat brain microvessel endothelial cell line. Neurosci Lett.

[74] Yang J, Mutkus LA, Sumner D, Stevens JT, Eldridge JC, Strandhoy JW (2001). Transendothelial permeability of chlorpyrifos in RBE4 monolayers is modulated by astrocyte-conditioned medium. Brain Res Mol Brain Res.

[75] Yang J, Aschner M (2003). Developmental aspects of blood-brain barrier (BBB) and rat brain endothelial (RBE4) cells as in vitro model for studies on chlorpyrifos transport. Neurotoxicology.

[76] Schiera G, Sala S, Gallo A, Raffa MP, Pitarresi GL, Savettieri G (2005). Permeability properties of a three-cell type in vitro model of blood-brain barrier. J Cell Mol Med.

[77] Fitsanakis VA, Piccola G, Aschner JL, Aschner M (2005). Manganese transport by rat brain endothelial (RBE4) cell-based transwell model in the presence of astrocyte conditioned media. J Neurosci Res.

[78] Li W, Ehrich M (2013). Transient alterations of the blood-brain barrier tight junction and receptor potential channel gene expression by chlorpyrifos. J Appl Toxicol.

[79] Alms D. Induction of multidrug transporters by antiepileptic drugs and known inducers and their transport in brain capillary endothelial cells of the blood-brain barrier of different species. Inaug.Diss. (Dr.rer.nat.) ed. Hannover: University of Veterinary Medicine; 2013. **(in German)**

[80] He Y, Yao Y, Tsirka SE, Cao Y (2014). Cell-culture models of the blood-brain barrier. Stroke.

