# Supplementary material for: Similarities and differences in the localization, trafficking, and function of P-glycoprotein in MDR1-EGFP-transduced rat versus human brain capillary endothelial cell lines
Source: Fluids Barriers CNS. 2021 Aug 3;18:36. doi: 10.1186/s12987-021-00266-z (PMC8330100; doi:10.1186/s12987-021-00266-z)
Supplement: Supplementary file 1 — Additional file 1. Influence of insert characteristics (Transwell® vs. ThinCert® and TC‐insert system) on monolayer formation and barrier integrity was assessed by comparison of transendothelial electrical resistance (TEER) and mannitol permeability of hCMEC/D3 and RBE4 cells. [file 12987_2021_266_MOESM1_ESM.pdf]

Additional file 1

Influence of insert characteristics (Transwell® vs. ThinCert® and TC-insert system) on monolayer formation and barrier integrity was assessed by comparison of transendothelial electrical resistance (TEER) and mannitol permeability of hCMEC/D3 and RBE4 cells. The pore diameter was 0.4 µm. Prior to seeding, the membranes were coated with rat-tail collagen type I. Data are presented as the mean ±SEM of 3-8 replicates. Significant differences between TEER values or mannitol permeability between RBE4 or hCMEC/D3 monolayers grown on the same insert were calculated by unpaired t-test and indicated by an asterisk ($P < 0.05$). Statistical differences between TEER values within hCMEC/D3 or RBE4 monolayers grown on different insert types calculated using one-way ANOVA (Bonferroni *posthoc* test) were indicated by # (vs. Greiner, 12-well) and † (vs. Corning, 6-well). Mannitol permeability of hCMEC/D3 or RBE4 monolayers was significantly different between all insert types ($P < 0.05$).

Cell type	TEER ($\Omega \cdot cm^2$)	Mannitol permeability		Insert type	Membrane material	Pore density ($pores/cm^2$)
		(nm/s)	(%/h)			
**hCMEC/D3-WT**	16.57 ± 1.06	53.76 ± 4.32	3.71 ± 0.13	12-well, Greiner, #665641	PET	$2 \cdot 10^6$
**RBE4-WT**	15.55 ± 1.22	59.58 ± 4.07	4.20 ± 0.30	12-well, Greiner, #665641	PET	$2 \cdot 10^6$
**hCMEC/D3-WT**	6.78 ± 0.18 *#†	198.19 ± 4.89	15.95 ± 0.30 *	12-well, Sarstedt, #83.3931.040	PET	$1 \cdot 10^8$
**RBE4-WT**	4.22 ± 0.18 #†	210.15 ± 3.52	19.18 ± 0.13	12-well, Sarstedt, #83.3931.040	PET	$1 \cdot 10^8$
**hCMEC/D3-WT**	8.21 ± 0.37	135.06 ± 3.72*	11.17 ± 0.36	12-well, Corning, #3460	PET	$4 \cdot 10^6$
**RBE4-WT**	7.09 ± 0.37 #†	153.89 ± 2.15	11.15 ± 0.24	12-well, Corning, #3460	PET	$4 \cdot 10^6$
**hCMEC/D3-WT**	14.01 ± 1.35 #	100.32 ± 2.02	14.76 ± 1.01	6-well, Corning, #3412	PC	$1 \cdot 10^8$
**RBE4-WT**	17.12 ± 1.56	113.41 ± 11.82	13.62 ± 0.80	6-well, Corning, #3412	PC	$1 \cdot 10^8$
