# Supplementary material for: Similarities and differences in the localization, trafficking, and function of P-glycoprotein in MDR1-EGFP-transduced rat versus human brain capillary endothelial cell lines
Source: Fluids Barriers CNS. 2021 Aug 3;18:36. doi: 10.1186/s12987-021-00266-z (PMC8330100; doi:10.1186/s12987-021-00266-z)
Supplement: Supplementary file 2 — Additional file 2. Cell morphology of RBE4 and hCMEC/D3 wildtype and MDR1-EGFP expressing cells. [file 12987_2021_266_MOESM2_ESM.pdf]

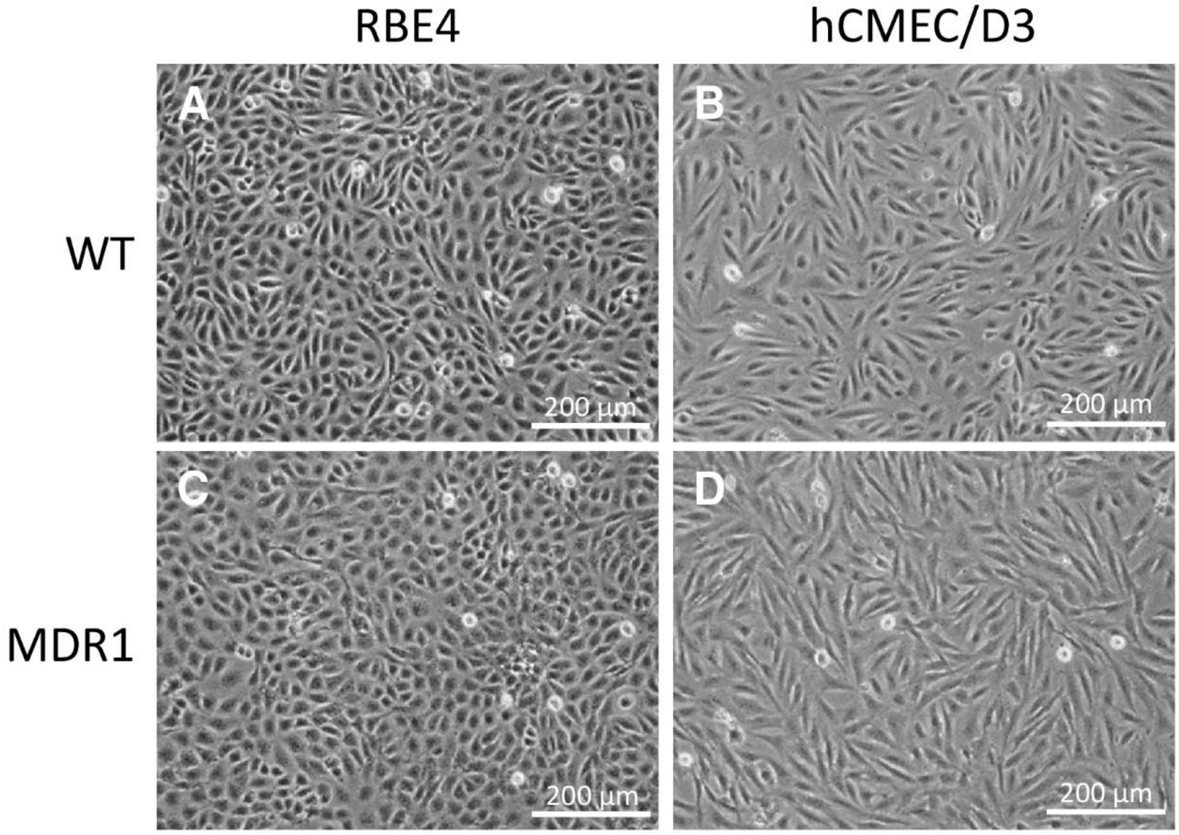

Additional file 2

Cell morphology of RBE4 and hCMEC/D3 wildtype and *MDR1*-EGFP expressing cells.

Cells were grown until confluency and cell morphology was analyzed by phase-contrast microscopy.
