# Supplementary material for: Similarities and differences in the localization, trafficking, and function of P-glycoprotein in MDR1-EGFP-transduced rat versus human brain capillary endothelial cell lines
Source: Fluids Barriers CNS. 2021 Aug 3;18:36. doi: 10.1186/s12987-021-00266-z (PMC8330100; doi:10.1186/s12987-021-00266-z)
Supplement: Supplementary file 3 — Additional file 3. Morphology of primary rat brain capillary endothelial cells (rBCECs) and immortalized LLC-MDR1 kidney epithelial cells. [file 12987_2021_266_MOESM3_ESM.pdf]

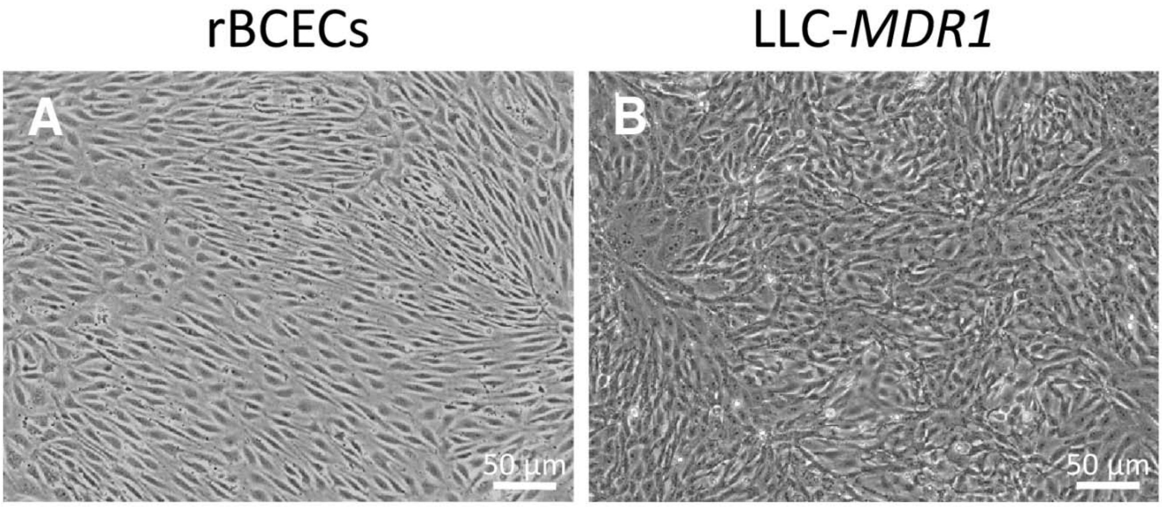

Additional file 3

Morphology of primary rat brain capillary endothelial cells (rBCECs) and immortalized LLC-*MDR1* kidney epithelial cells. Cells were grown until confluency and cell morphology of the primary culture of rBCECs (A) and the epithelial cell line LLC-*MDR1* (B) were depicted by phase-contrast microscopy.
