# Supplementary material for: Similarities and differences in the localization, trafficking, and function of P-glycoprotein in MDR1-EGFP-transduced rat versus human brain capillary endothelial cell lines
Source: Fluids Barriers CNS. 2021 Aug 3;18:36. doi: 10.1186/s12987-021-00266-z (PMC8330100; doi:10.1186/s12987-021-00266-z)
Supplement: Supplementary file 4 — Additional file 4. P-glycoprotein localization in MDR1-EGFP expressing RBE4 and hCMEC/D3 cells. [file 12987_2021_266_MOESM4_ESM.pdf]

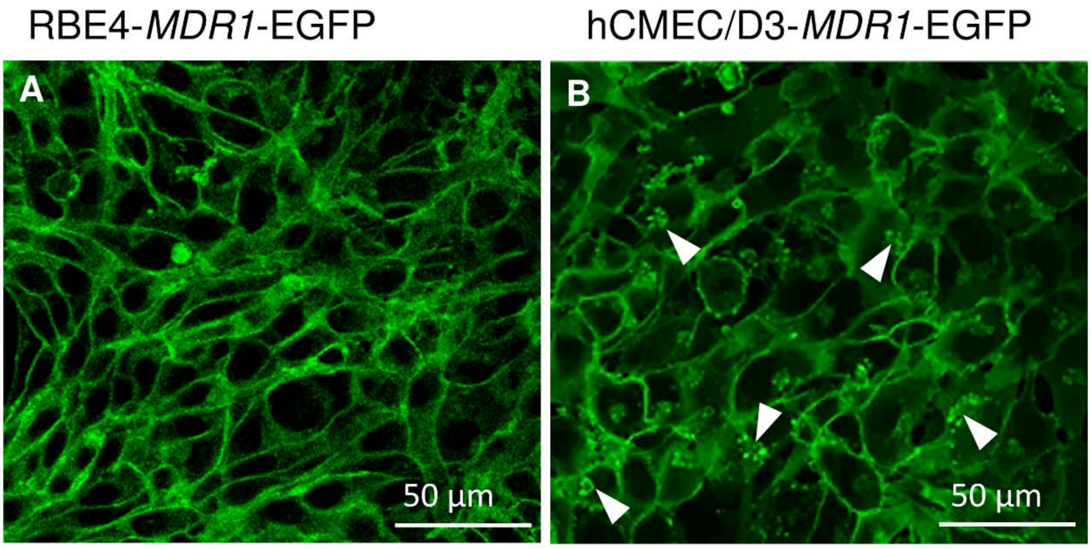

Additional file 4

P-glycoprotein localization in *MDR1*-EGFP expressing RBE4 and hCMEC/D3 cells.

Cells were grown until confluency and Pgp-EGFP (green) localization was assessed using confocal laser scanning fluorescence microscopy and live-cell imaging. Note the higher cytoplasmic expression of Pgp-EGFP in lysosome-like vesicles in hCMEC/D3 (arrows) vs. RBE4 cells, in which such vesicles were hardly visible.
