# Supplementary material for: Similarities and differences in the localization, trafficking, and function of P-glycoprotein in MDR1-EGFP-transduced rat versus human brain capillary endothelial cell lines
Source: Fluids Barriers CNS. 2021 Aug 3;18:36. doi: 10.1186/s12987-021-00266-z (PMC8330100; doi:10.1186/s12987-021-00266-z)
Supplement: Supplementary file 5 — Additional file 5. Pgp-EGFP-positive intracellular vesicles in RBE4 and hCMEC/D3 cells before and after exposure to doxorubicin (DOXO). [file 12987_2021_266_MOESM5_ESM.pdf]

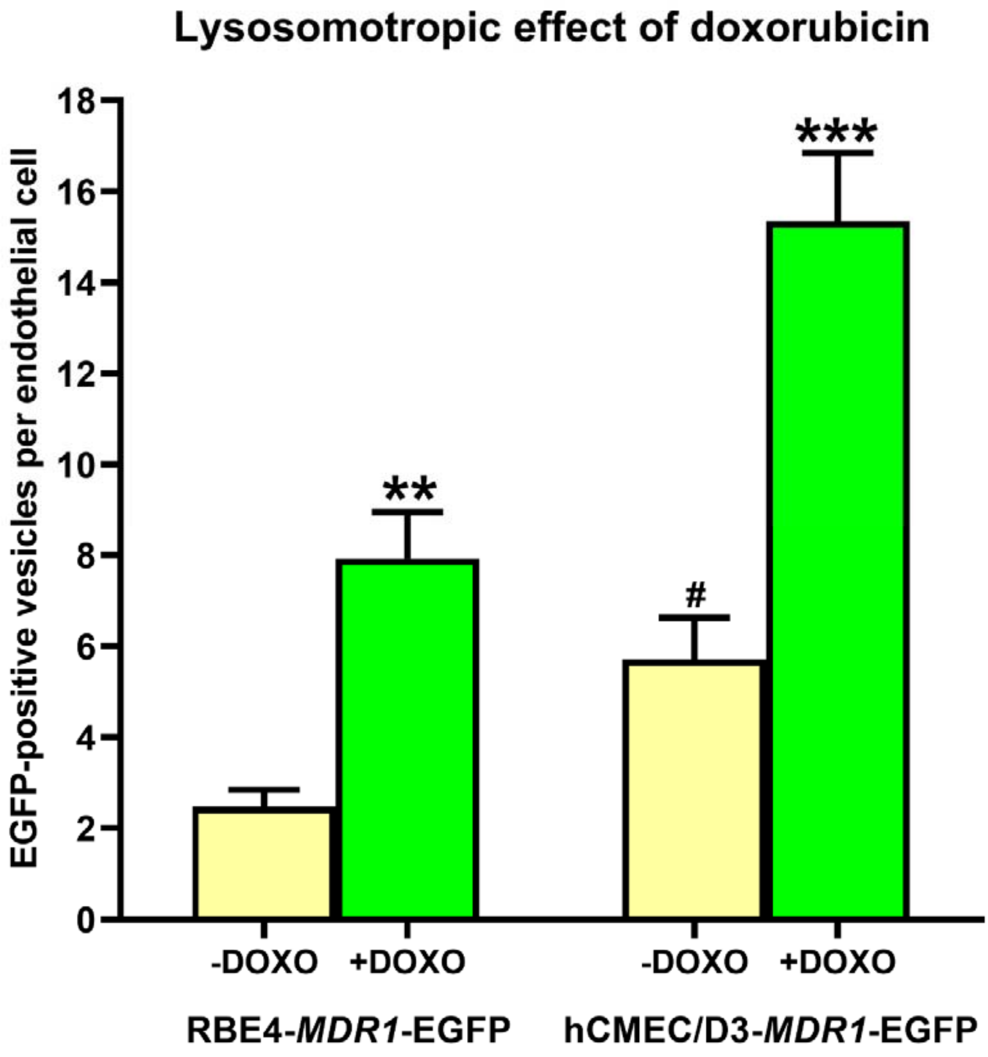

Additional file 5

Pgp-EGFP-positive intracellular vesicles in RBE4 and hCMEC/D3 cells before and after exposure to doxorubicin (DOXO). Pgp-EGFP-positive intracellular vesicles were counted in 60-81 RBE4 cells and 46 hCMEC/D3 cells; data are shown as mean ± SEM. Data were analyzed by nonparametric one-way ANOVA (Kruskal-Wallis test) followed by Dunn's multiple comparisons test. For each cell line, significant differences in the number of Pgp-EGFP-positive intracellular vesicles before vs. 24 h after DOXO treatment are indicated by asterisks (**P<0.001; ***P<0.0001). Significant differences in the number of Pgp-EGFP-positive intracellular vesicles in RBE4 vs. hCMEC/D3 cells before DOXO treatment are indicated by the hash sign (#P<0.05).
