# Supplementary material for: Similarities and differences in the localization, trafficking, and function of P-glycoprotein in MDR1-EGFP-transduced rat versus human brain capillary endothelial cell lines
Source: Fluids Barriers CNS. 2021 Aug 3;18:36. doi: 10.1186/s12987-021-00266-z (PMC8330100; doi:10.1186/s12987-021-00266-z)
Supplement: Supplementary file 6 — Additional file 6. Indirect staining of Pgp in MDR1-EGFP transduced RBE4 and hCMEC/D3 cells colocalizes with the Pgp-EGFP fluorescence signal. [file 12987_2021_266_MOESM6_ESM.pdf]

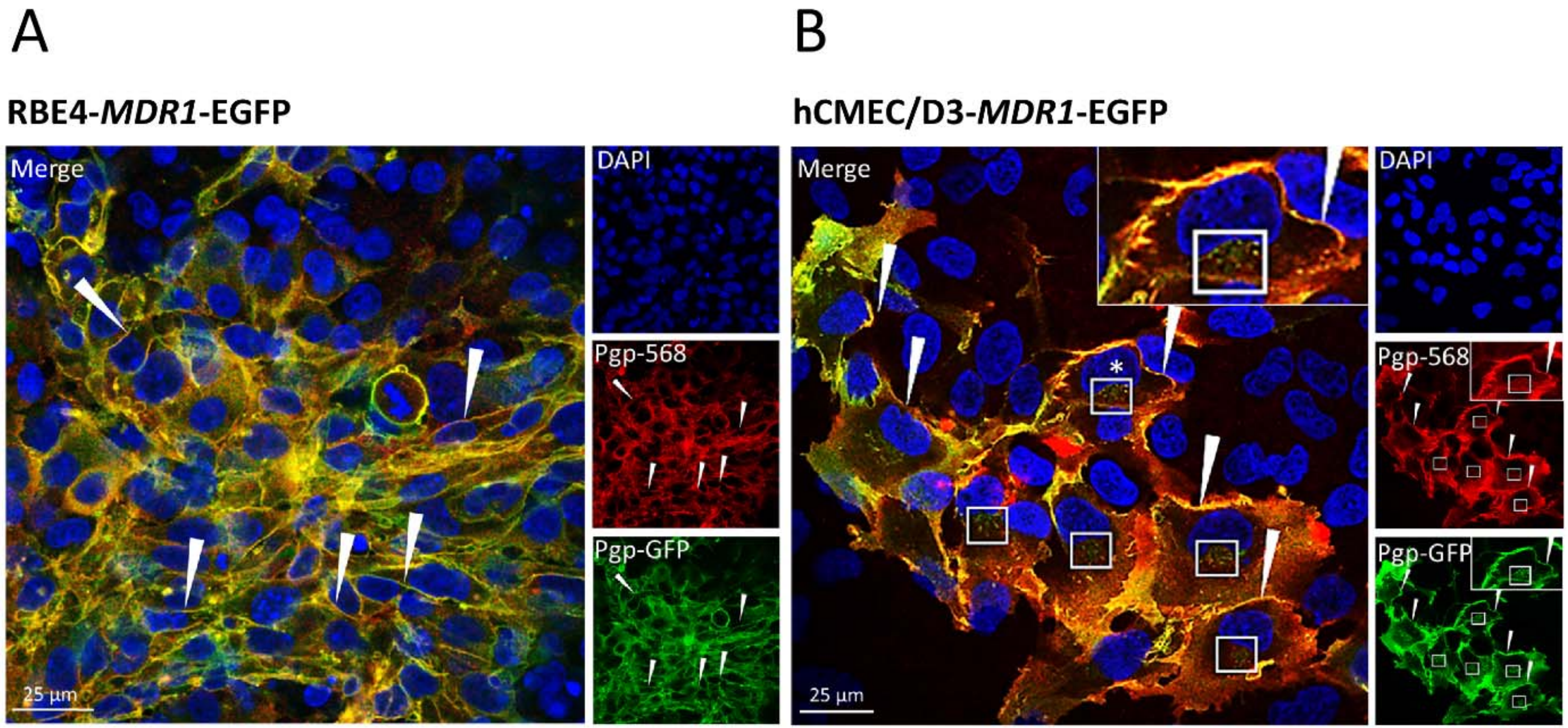

Additional file 6

Indirect staining of Pgp in *MDR1*-EGFP transduced RBE4 and hCMEC/D3 cells colocalizes with the Pgp-EGFP fluorescence signal.

Colocalization of Pgp-EGFP signal (green) and indirect Pgp staining (red: Alexa 568) after fixation of the confluent RBE4-*MDR1*-EGFP (A) and

hCMEC/D3-*MDR1*-EGFP cells (B) with paraformaldehyde substantiates that the GFP signal reflects cellular Pgp localization of the Pgp-EGFP protein overexpressed in the cells and is not indicative of cleaved EGFP tag. Pgp plasma membrane localization is highlighted by arrowheads and intracellular vesicular Pgp localization in hCMEC/D3-*MDR1*-EGFP cells by boxes. In the upper right corner of B, a hCMEC-*MDR1*-EGFP cell with intracellular vesicular Pgp localization is magnified. Cell nuclei are visible by counterstaining with DAPI (blue).
