# Supplementary material for: Similarities and differences in the localization, trafficking, and function of P-glycoprotein in MDR1-EGFP-transduced rat versus human brain capillary endothelial cell lines
Source: Fluids Barriers CNS. 2021 Aug 3;18:36. doi: 10.1186/s12987-021-00266-z (PMC8330100; doi:10.1186/s12987-021-00266-z)
Supplement: Supplementary file 7 — Additional file 7. Pgp localization in RBE4-WT cells by indirect Pgp staining. [file 12987_2021_266_MOESM7_ESM.pdf]

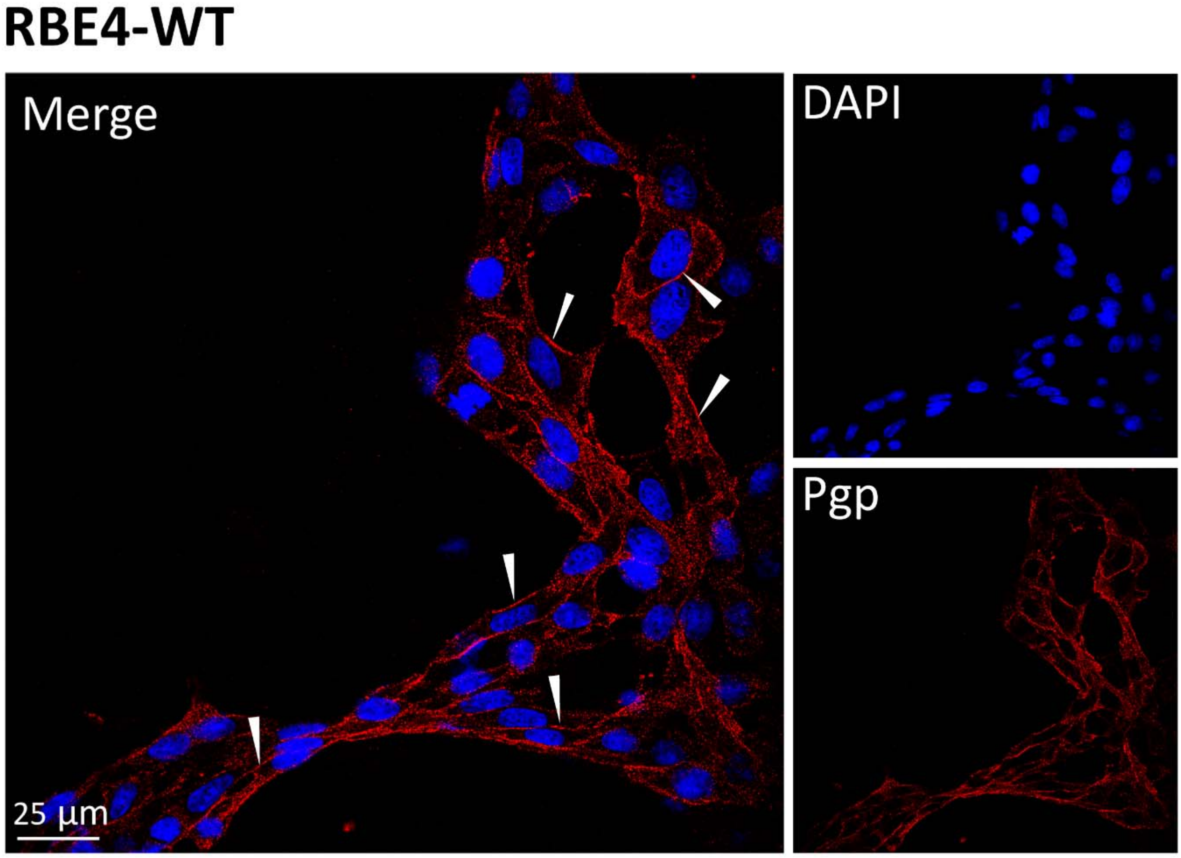

Additional file 7

Pgp localization in RBE4-WT cells by indirect Pgp staining. RBE4 cells were seeded on coverslips and indirectly stained for Pgp after fixation. As assessed by confocal laser scanning microscopy Pgp (red) was located on the cell surface (arrowheads) of the RBE4-WT cells. Similar to the *MDR1*-EGFP-transduced RBE4 cells (Fig. 1A), no Pgp-positive intracellular vesicles were observed in the RBE4-WT cells. Cell nuclei were visualized by DAPI staining (blue).
